# Region-specific transcriptional signatures of brain aging in the absence of neuropathology at the single-cell level

**DOI:** 10.1038/s41514-026-00391-9

**Published:** 2026-05-13

**Authors:** Monica E. Mesecar, Megan F. Duffy, Dominic J. Acri, Jinhui Ding, Rebekah G. Langston, Syed I. Shah, Mike A. Nalls, Xylena Reed, Sonja W. Scholz, D. Thad Whitaker, Pavan K. Auluck, Stefano Marenco, Alex R. DeCasien, J. Raphael Gibbs, Mark R. Cookson

**Affiliations:** 1 Cell Biology and Gene Expression Section, Laboratory of Neurogenetics, National Institute on Aging, National Institutes of Health, Bethesda, MD USA; 2 Computational Biology Group, Laboratory of Neurogenetics, National Institute on Aging, National Institutes of Health, Bethesda, MD USA; 3 DataTecnica LLC, Washington, DC USA; 4 Center for Alzheimer’s and Related Dementias, National Institutes of Health, Bethesda, MD USA; 5 Neurodegenerative Diseases Research Section, National Institute of Neurological Disorders and Stroke, Bethesda, MD USA; 6 Department of Neurology, Johns Hopkins University Medical Center, Baltimore, MD USA; 7 Human Brain Collection Core, Division of Intramural Research, National Institute of Mental Health, NIH, Bethesda, MD USA; 8 Computational and Evolutionary Neurogenomics Unit, Laboratory of Neurogenetics, National Institute on Aging, National Institutes of Health, Bethesda, MD USA

**Keywords:** Genetics, Neurology, Neuroscience

## Abstract

As age is a significant risk factor for multiple neurodegenerative diseases, investigating normal brain aging may help identify molecular events contributing to increased disease risk over time. Single-nucleus RNA sequencing (snRNA-seq) enables analysis of gene expression changes within specific cell-types, offering insights into the molecular mechanisms underlying aging. However, most brain aging snRNA-seq datasets use age-matched controls from studies focused on pathology and sample cortical regions. Therefore, there is a need to investigate non-pathological aging within brain regions vulnerable to age-related diseases. We report a snRNA-seq study of 6 young (20–30 years) and 7 aged (60–85 years) individuals encompassing four different brain regions: the entorhinal cortex, middle temporal gyrus, subventricular zone, and putamen. We captured over 150,000 nuclei representing 10 broad cell-types. Region- and cell-type-specific differential expression analyses identified over 8000 age-associated genes. Notably, within a given cell-type, most of these associations were region-specific. Functional enrichment analyses of gene sets for each cell-type-region subgroup reflected multiple hallmarks of aging, including: proteostasis, interactions with cytokines, vesicular trafficking, metabolism, inflammation, metal ion homeostasis, and cellular senescence. Overall, our findings suggest that unique cell-types exhibit distinct transcriptional aging profiles both at the cell-type level and across different brain regions.

## Introduction

Age is a common risk factor for multiple neurodegenerative diseases (NDDs), including Alzheimer’s disease, Parkinson’s disease, and amyotrophic lateral sclerosis (ALS)^1^. These age-related neurodegenerative diseases each have patterns of regional susceptibility. However, it remains unclear whether these regional differences are intrinsically linked to disease-causing mechanisms or reflective of global changes in brain health as an individual ages. Studies of NDDs often use age-matched controls without similar neuropathology to control for age as a source of variability. However, there is still a critical need to understand which cell-type- and region-specific effects arise specifically in individuals without NDDs during normal brain aging.

Prior studies have used bulk transcriptomic and epigenomic analyses to explore the effects of aging on the human brain. Major themes emerging from these investigations include loss of synaptic gene expression and acquisition of inflammatory signaling networks^2^. Our previous RNA-sequencing (RNA-seq) analyses of the human dorsolateral prefrontal cortex (dlPFC) identified networks of gene expression changes with age, including loss of neuronal genes^3^. For example, there was a strong decrease in *SST*, the gene encoding the peptide neurotransmitter somatostatin, which defines one of the major subtypes of inhibitory neurons in the human cerebral cortex.

Although the literature suggests that robust changes in gene expression correlate with age, several aspects of the data remain difficult to interpret. For example, loss of neuronal markers could reflect a loss of neurons or changes in gene expression among neurons. Recent development of single-cell methods allows for the identification of unique cell-types and the interrogation of gene expression within each population. However, to date, single-cell studies of postmortem human brain tissue have focused primarily on the cerebral cortex^4–7^. Therefore, it is unknown whether cell-type-specific aging signatures are similar across different brain regions. Given the known effects of non-pathological aging on the human cerebral cortex, we aimed to investigate which age-effects were regionally distinct versus shared broadly across regions that have shown vulnerability to age-related diseases.

We included two cortical areas that are susceptible to Alzheimer’s disease (AD) pathology: the entorhinal cortex (EC), which is affected early in the disease, and the middle temporal gyrus (MTG), which is affected at later stages^8,9^. We also included the putamen (PUT), which is affected by Huntington’s disease (HD)^10,11^ and is the target of dopaminergic neurons that are lost in Parkinson’s disease (PD)^12–14^ and the subventricular zone (SVZ), which is permissive for neurogenesis during development, although whether this remains true in the adult human brain is still contested^15–17^. By curating a cohort of young (20–30 years old) and aged (60–85 years old) individuals who lacked evidence for NDD-related pathology, we aimed to document age-related transcriptional changes across different cell types and regions. We did not observe statistically significant differences in cell-type proportions across age with this sample size. The lack of proportion changes in this study may be due to modest sample sizes combined with limited precision in microfluidic-based single-cell assays^18,19^. By performing differential expression, we did find large effects of age on the expression of specific genes per cell-type. Notably, the vast majority of age-associated differentially expressed genes (aDEGs) were unique to specific regions or cell types. We find that aDEGs within specific cell-type–region subgroups are associated with previously reported hallmarks of aging^20,21^, including proteostasis, interactions with cytokines, vesicular-trafficking, metabolism, the immune system and inflammation, metal ion homeostasis, and senescence. Importantly, we show that the effect of aging is region-specific even within cell types that are consistently present in cortical and subcortical regions. Taken together, our data serve as a resource for those studying age-related processes in the absence of disease-associated neuropathology.

## Results

### Cell-type-specific profiles of aging across multiple human brain regions

**Fig. 1:**
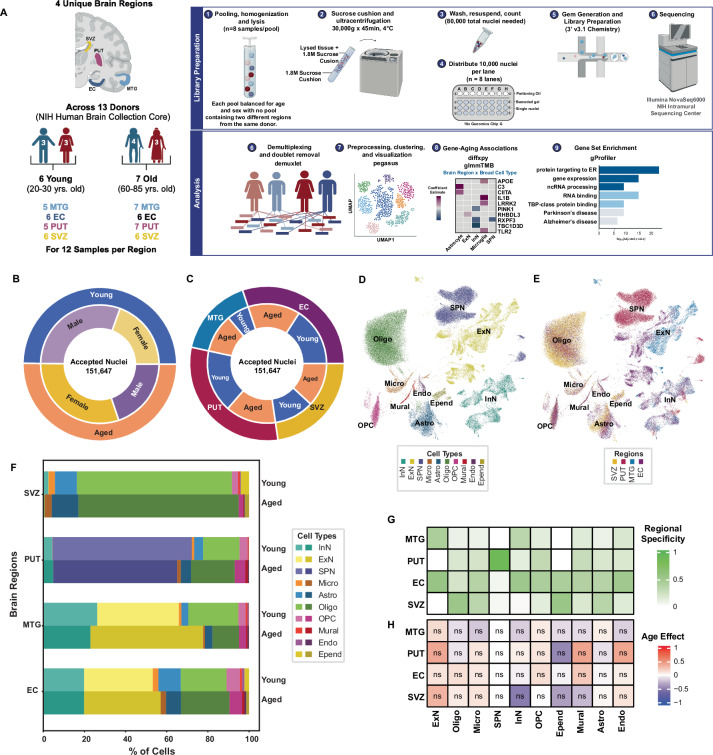
A single-cell dataset for human brain aging across cell types and regions. **A** An outline of the single-nucleus RNA sequencing study design, including sample selection and workflow for investigating age-related differentially expressed genes (aDEGs) across 4 human brain regions: Entorhinal cortex (EC), Middle Temporal Gyrus (MTG), Putamen (PUT), and Subventricular Zone (SVZ). The final aDEG set was those with an FDR-BH adjusted *p*-value less than 0.05. Graphics created with Biorender.com. **B** Distribution of 151,647 accepted nuclei recovered per condition, shown by age group and sex, demonstrates approximately even recovery across categories. **C** Nuclei distribution shown by brain region and age group (see also Supplementary Data file 2). **D** Clustering of 151,647 nuclei using the Leiden algorithm (see “Methods”) resulted in 25 clusters representing 10 unique broad cell-types. Clusters are colored and labeled according to broad cell-type annotation and **E** brain region of origin. Initial broad cell-type annotations were made using a combination of *Pegasus*-determined cluster markers (see “Methods”) and relative expression levels of canonical marker genes for various known CNS cell-types (Supplementary Fig. 2 and Supplementary Data files 3, 4) This process revealed 3 neuronal populations (InN: inhibitory neurons, ExN: excitatory neurons, SPN: spiny projection neurons), 3 glial populations (Micro: microglia, Astro: astrocytes, Epend: ependymal cells), 2 oligodendrocyte-lineage populations (OPC: oligodendrocyte precursor cells, Oligo: oligodendrocytes), 1 endothelial (Endo), and 1 mural cell population each. **F** The proportions of 10 unique cell-types found across the SVZ, PUT, MTG, and EC by age group. From the accepted 151,647 nuclei, percentages were calculated using raw cell counts within a given region by age subset (*n* = 5–7 individuals per region per age group). **G** Regional specificity of cell counts shows even distribution of cell-types across all regions of interest, except SPN, which are known to be restricted to midbrain regions^23^. Regional specificity was calculated as a proportion of the number of a particular cell-type in the region of interest (ROI) over the total number of cells of that type (e.g., # ExN_MTG/# ExN_total). **H** Age-effect on cell-type proportions reveals no effect (FDR > 0.05) in any cell-type across all 4 ROIs. Relative cell-type proportions were compared with a t-test, and FDR-BH corrected to assess significance.

We aimed to investigate how gene expression profiles of different brain cell populations change with age across distinct brain regions. To this end, postmortem human brain tissue samples were obtained from four brain regions, including the entorhinal cortex (EC), middle temporal gyrus (MTG), subventricular zone (SVZ), and putamen (PUT). For 13 individuals, including six younger (20–30 years old) and seven older (60–85 years old) donors, 12 tissue samples were obtained for each of the four regions for a total of 48 samples. Donor groups were balanced for sex and tissue number (see Supplementary Fig. 1 and Supplementary Data file 1 for donor and tissue demographics). Nuclei were isolated from the selected tissue samples, processed, and sequenced as described in Methods and shown schematically in Fig. 1A.

After quality control and pre-processing, we captured 151,647 nuclei, with similar recovery across age groups (*n* = 75,547 young; 76,100 aged) and sexes (*n* = 76,084 male; 75,563 female). Nuclei counts and relative nuclei proportions by sex within each age group are shown in Fig. 1B. By region, the nuclei distribution was 45,688 EC; 25,026 MTG; 34,444 SVZ; and 46,489 PUT. Relative nuclei proportions by age within each region are shown in Fig. 1C. Raw nuclei counts by age and sex for each cell-type and region are provided in Supplementary Data file 2.

Using a combination of computed cluster marker genes and known CNS cell-type marker genes (Supplementary Data file 3 and 4), 25 distinct clusters were annotated into 10 broad cell-types (Supplementary Fig. 2), including inhibitory neurons (InN), excitatory neurons (ExN), spiny projection neurons (SPN), oligodendrocyte precursor cells (OPC), oligodendrocytes (Oligo), astrocytes (Astro), microglia (Micro), ependymal cells (Epend), endothelial cells (Endo), and mural cells (Fig. 1D; see full cell taxonomy in Supplementary Data file 5). As expected, we found that some cell types, such as microglia and oligodendrocyte precursor cells, were uniformly represented across all regions, while others, such as spiny projection neurons and excitatory neurons, showed greater regional specificity (Fig. 1E). These observations indicate that we were able to generate a valid dataset to examine age-related changes in gene expression across multiple regions of the human brain.

### Cell-type proportions show no change across age groups

We investigated the effect of regional biases and age on cell-type proportions (Fig. 1F; see “Methods” for calculation). In total, 17,733 nuclei were annotated as inhibitory neurons (*n* = 8734 young; 8999 aged), and we found that these nuclei predominate in the MTG (26.23% young; 22.94% aged) and EC (19.87% young; 19.89% aged; Fig. 1F). Similarly, excitatory neurons (*n* = 11,757 young; 16,726 aged) were also recovered at high levels in the MTG (39.69% young; 54.81% aged) and EC (33.34% young; 37.17% aged; Fig. 1F). A total of 29,967 cells were labeled as spiny projection neurons (17,473 young; 12,494 aged), and all were localized to the PUT (Fig. 1F). There were initially nuclei annotated as spiny projection neurons (SPN) in the MTG, EC, and SVZ(Supplementary Fig. 3A–C), a neuronal subtype known to be specific to the caudate and putamen^22^. We considered such assignments to be unreliable and likely due to a combination of dissection variability (sampling from adjacent ganglia during dissection of the SVZ) or due to clustering of other inhibitory neurons, of which SPNs are a specialized subtype (Supplementary Fig. C–E). Therefore, any non-PUT cells initially annotated as SPN were re-classified as “Other” (*n* = 3545 cells) and excluded from further analyses. While the 50,129 cells (*n* = 24,545 young; 25,584 aged) annotated as oligodendrocytes were present in all regions, the majority originated from the SVZ (75.35% young; 77.91% aged; Fig. 1F). These regional distinctions are further quantified via a regional specificity value (see “Methods”) that show that all other cell-types were uniformly distributed across regions (Fig. 1G). We report no statistically significant effect of age to any cell-type proportion across all regions (FDR-BH adj. *P*-value > 0.05 for all; Fig. 1H; see “Methods” for proportion analysis). Noting that the sample size is relatively modest, this does not allow us to fully exclude that there may be some changes in cell number. Based on these observations, we therefore tested the effect of age on gene expression within cell-type by region subgroups.

### Thousands of genes are differentially expressed across age groups

Gene sets were tested for age-related differential expression in a two-step process. We performed an initial t-test to identify the strongest effect size candidates, followed by a generalized linear mixed model with Tweedie distribution to more rigorously identify aDEGs (see “Methods”), followed by Benjamini–Hochberg False Discovery Rate (FDR-BH) correction for this more limited multiple testing. Across all cell-types, 8872 unique genes were age-associated via linear model (Supplementary Data file 6). The majority of aDEGs were found to be protein-coding, with the remaining being long non-coding RNAs (Supplementary Fig. 4 and Supplementary Data file 6). While the number and age-effect on aDEGs are region- and cell-type-specific, the majority of aDEGs showed decreased expression with age (Supplementary Fig. 5 and Supplementary Data file 6).

Previous studies have observed that longer genes are more likely to be downregulated with age, which is suggested to occur due to longer genes having a greater propensity for accumulating DNA damage over time^23,24^. However, these studies reflect bulk expression in non-brain tissues from mice. Therefore, the associations found in these studies may not translate to human brain tissue nor be consistent across cell types or brain regions. In a recent single-cell study^7^, shorter genes were found to be differentially impacted by the aging process while longer genes maintained their expression, but this finding was limited to neuronal cell types in the prefrontal cortex. In order to further interrogate the relationship between gene length and age-related gene expression changes, we compared the length distributions of aDEGs to genes that did not show age association in the current analysis at both broad and cell-type-by-region levels.

Using genomic-span information from our reference genome (GRCh38-2020-A), we found the median to be 14,454 bases (4.16 after log10-transformation; Supplementary Fig. 6A). Next, we compared the lengths of all aDEGs (median log10 base pairs = 4.67) versus all other genes (median log10 base pairs = 4.40) and found that on average, aDEGs were significantly longer than genes without age association (Welch’s two sample t-test; *t*_15,842_ = 29.914, *p*-value < 2.2E-16), (Supplementary Fig. 6B). A similar relationship between gene length and whether the gene was identified as an aDEG was generally seen across all cell type by region combinations (compared via Wilcoxon Rank-Sum test; Supplementary Fig. 6C and Supplementary Data file 7). The one exception was within inhibitory neurons, where non-aDEGs were found to be significantly longer than aDEGs within three regions (MTG *p* = 1.05E-10; EC *p* = 8.66E-17 ; SVZ *p* = 1.76E-3). Collectively, these results support prior data that longer genes are more likely to show age associations compared to shorter genes, but that there are specific cell types where that association may be inverted. Based on these observations, we next examined how aDEGs were distributed in different cell types.

### Inhibitory neurons demonstrate the greatest age effect among the neuronal cell-types across regions, including dysregulation of protein synthesis

**Fig. 2:**
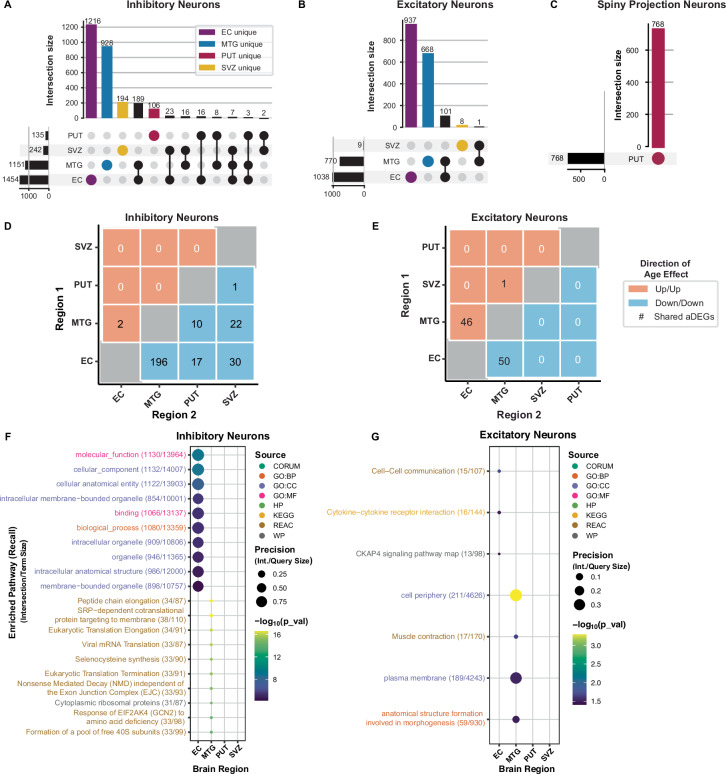
Inhibitory neurons exhibit the largest number of age-associated differentially expressed genes (aDEGs) amongst neuronal cell-types. Regional distribution and overlap of neuronal cell-type aDEGs indicate that the majority of aDEGs are unique to a particular region. **A** Inhibitory neurons yielded the largest number of aDEGs, with the most being found in the EC. **B** Excitatory neurons had fewer total aDEGs but followed similar trends. **C** Spiny projection neurons are known to be biologically distinct from the putamen^23^, so they were only reported for this region (see Fig. 1H). Pairwise comparison of shared aDEGs within neuronal cell-types suggests similar signatures between the EC and MTG. Heatmap values representing count of aDEGs indicate the number of aDEGs changing in the same direction (concordance)—either increasing (positive, red) or decreasing (negative, blue)—in both of the indicated regions. **D** Inhibitory neurons showed 196 negatively concordant aDEGs between the EC and MTG. **E** Excitatory neurons showed 96 concordant aDEGs between the EC and MTG, with 46 positive and 50 negative. Top 10 significantly enriched pathways (FDR < 0.05) per brain region for neuronal cell-type aDEGs. Functional enrichments calculated in gprofiler2 with cell-type by region background correction (see “Methods”). **F** Inhibitory neurons exhibited functional enrichments for pathways involving cellular components and translation/protein synthesis pathways. **G** Excitatory neurons exhibited functional enrichments for pathways involving cellular components as well as cell communication and signaling.

Within neuronal cell-types, inhibitory neurons were the most affected by age, with 2708 aDEGs. Of these inhibitory neuron aDEGs, 2444 were specific to a single region, with the majority being in the EC (*n* = 1216; Fig. 2A). In excitatory neurons, we identified 1715 aDEGs. Of these, 1613 were contained within a single region, with the most being found in the EC (*n* = 937; Fig. 2B). In PUT-specific spiny projection neurons, we found 768 aDEGs (Fig. 2C). All aDEGs for these cell types can be found in Supplementary Data file 6.

The proportion of aDEGs that were shared across regions for neurons ranged from 1.1% to 11.4%. The majority of these shared-aDEG relationships occurred between two brain regions (pairwise). Generally, the direction of age-effect was found to be concordant across regions and cell-types. In inhibitory neurons, the most striking of these relationships were 196 aDEGs shown to decrease in both the EC and MTG (negatively concordant; Fig. 2D). In contrast, for excitatory neurons, there were approximately even numbers of aDEGs that were increased (positively concordant; *n* = 46) or decreased with age (negatively concordant; *n* = 50) across both the EC and MTG (Fig. 2E). aDEGs that showed increased expression with age in one region but decreased expression in another (discordant relationships) were present but much fewer in number relative to concordant relationships (Supplementary Fig. 7). In addition, while there were instances of aDEGs being shared across more than two regions, these were relatively infrequent.

There were 96 enriched pathways across all neuronal cell-types, with 88 observed in inhibitory neurons, all of which were restricted to the EC (*n* = 20) or MTG (*n* = 68; Supplementary Data file 8). Within the EC, the top inhibitory neuron pathways were related to cellular anatomy and subcellular components (e.g., organelles; Fig. 2F). Within the MTG, significant inhibitory neuron pathways related to translational processes, protein synthesis, and ribosomal structures (Fig. 2F). Excitatory neurons had seven total enriched pathways, all of which were also restricted to the EC (*n* = 3) or MTG (*n* = 4; Supplementary Data file 8). In the EC, we observed excitatory neuron pathways related to cell-cell communication, particularly relevant to cytokine receptor interactions and *CKAP4* signaling (Fig. 2G). In the MTG, excitatory neuron pathways related to cellular morphology—specifically the plasma membrane—and muscle contraction (Fig. 2G). PUT-specific spiny projection neurons were enriched for “*MECP2* and associated Rett syndrome” (WikiPathways: WP3584; Supplementary Data file 8). Taken together, these observations suggest distinct transcriptional changes across neuronal cell types and regions with aging.

### Oligodendrocyte-lineage cells exhibit regionally distinct aging signatures related to neuronal structure and vesicular transport

**Fig. 3:**
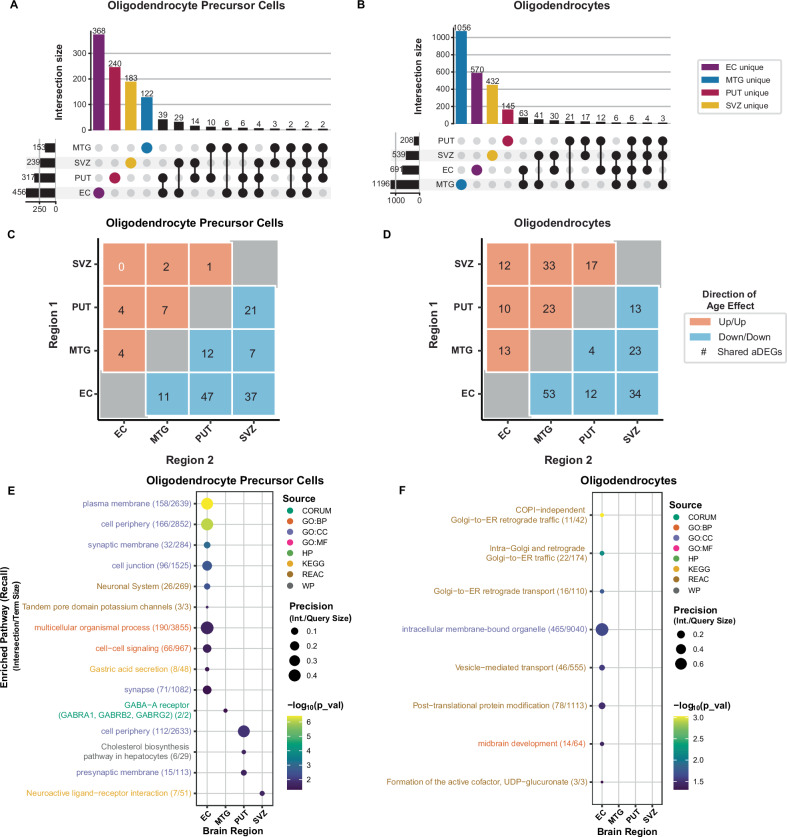
Oligodendrocyte-lineage cell aDEGs are enriched for neuronal cellular component and vesicular transport pathway. Regional distribution and overlap of oligodendrocyte lineage cell-type aDEGs indicate that the majority of aDEGs are unique to a particular region. Of the oligodendrocyte-lineage cell-types, **A** oligodendrocyte precursor cells had its greatest proportion of aDEGs in the EC. **B** Mature oligodendrocytes had far more aDEGs, with the majority in the MTG. Pairwise comparison of shared aDEGs within oligodendrocyte lineage cells suggests variation between oligodendrocyte precursor cells and mature oligodendrocytes. Heatmap values representing count of aDEGs indicate the number of aDEGs changing in the same direction (concordance)--either increasing (positive, red) or decreasing (negative, blue)–in both of the indicated regions. **C** Oligodendrocyte precursor cells concordant aDEGs were largely in the negative direction. **D** Mature oligodendrocytes had a more balanced split between positively and negatively concordant aDEGs. Top 10 significantly enriched pathways (FDR < 0.05) per brain region for oligodendrocyte-lineage cell-type aDEGs. Functional enrichments calculated in gprofiler2 with cell-type by region background correction (see Methods). **E** OPCs showed enriched pathways for various neuronal cellular components and receptor pathways. **F** Mature oligodendrocytes showed enriched pathways related to vesicular transport.

In oligodendrocyte precursor cells (OPCs), there were 1030 aDEGs (Supplementary Data file 6), with the majority of these aDEGs restricted to a single region (*n* = 913), particularly the EC (Fig. 3A). Mature oligodendrocytes yielded 2406 aDEGs, and 2203 of these were specific to a single region, with just under half (*n* = 1056) being unique to the MTG (Fig. 3B and Supplementary Data file 6). These findings suggest that oligodendrocyte precursor cells and mature oligodendrocytes show regionally distinct aging profiles. These distinct aging profiles are also observed when examining concordance in expression direction for oligodendrocyte-lineage aDEGs shared pairwise between regions. In OPCs, the aDEGs shared across regions were concordant in a negative direction, and the regions exhibiting the most aDEG overlap are the EC and the PUT (*n* = 47), followed by the EC and SVZ (*n* = 37; Fig. 3C). In contrast, for mature oligodendrocytes, there was a more even distribution between positively concordant and negatively concordant instances with the regions of greatest overlap being the EC and MTG (*n* = 53, negatively concordant) and the SVZ and MTG (*n* = 33; Fig. 3D). Discordant relationships are presented in Supplementary Fig. 7.

Across oligodendrocyte-lineage cells, 24 enriched pathways were identified (*n* = 16 OPC; *n* = 8 Oligo; Supplementary Data file 8.). In oligodendrocyte precursor cells, significantly enriched pathways were found across all four regions, with the most being found in the EC (*n* = 10; Fig. 3E and Supplementary Data file 8). Of these EC-OPC pathways, several relate to neuronal cell structures (e.g., plasma membrane, cell periphery, synaptic membrane, cell junction, neuronal system, potassium channels, and synapse). PUT-OPC aDEGs also showed enriched pathways related to the cell periphery and the presynaptic membrane. Oligodendrocyte precursor cells in the MTG and SVZ were enriched for the GABA-A receptor (CORUM: 5809) and the neuroactive ligand-receptor interaction (KEGG: 04080), respectively (Fig. 3E and Supplementary Data file 8). In mature oligodendrocytes, enriched pathways were related to Golgi-to-ER retrograde transport of vesicles and were restricted to the EC (Fig. 3F and Supplementary Data file 8).

### Astrocytic and microglial cell aDEGs are found largely in the SVZ and EC and exhibit diverse functional enrichments

**Fig. 4:**
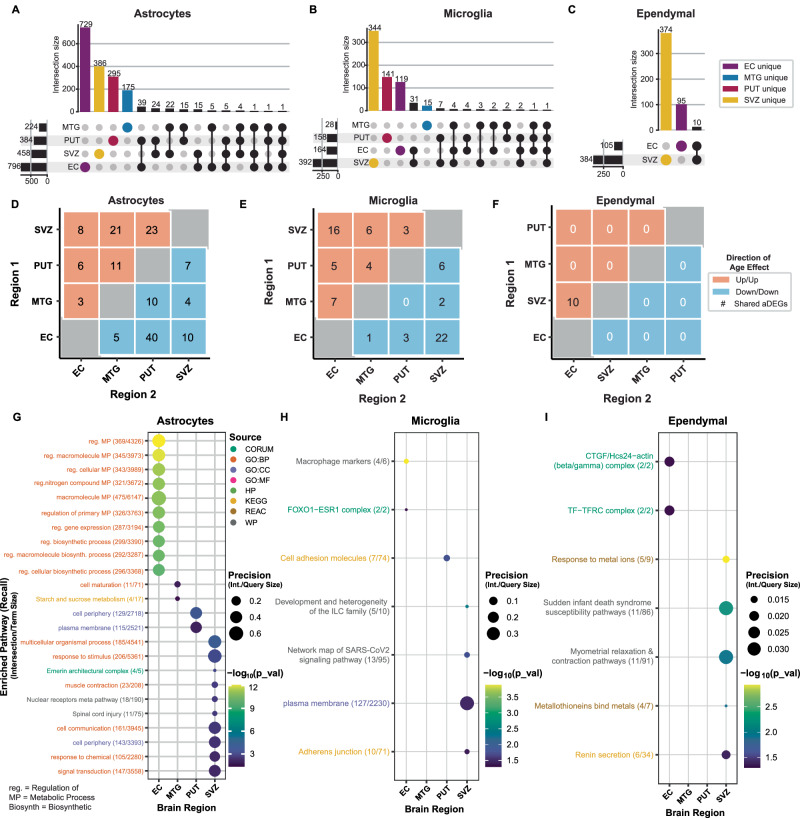
SVZ and EC account for the largest proportion of glial cell aDEGs and exhibit diverse functional enrichments. Regional distribution and overlap of glial cell-type aDEGs indicate that the majority of aDEGs are unique to a particular region. **A** Astrocytes yielded the most aDEGs of the glial cell-types with the greatest number being found in the EC. **B** Microglia aDEGs were largely found in the SVZ. **C** Ependymal cells had the fewest aDEGs of the glial cell types and were found to be contained to the EC and SVZ. Pairwise comparison of shared aDEGs within glial cell-types suggests similar signatures between the EC and SVZ. Heatmap values representing count of aDEGs indicate the number of aDEGs changing in the same direction (concordance)—either increasing (positive, red) or decreasing (negative, blue)—in both of the indicated regions. **D** Astrocytes had a relatively large number of negatively concordant aDEGs across the EC and PUT. **E** Microglia showed their greatest numbers of concordant aDEGs between the SVZ and EC in both the positive and negative directions. **F** Ependymal cell concordant aDEGs were solely positive and contained to the SVZ and EC. Top 10 significantly enriched pathways (FDR < 0.05) per brain region for glial cell-type aDEGs. Functional enrichments calculated in gprofiler2 with cell-type by region background correction (see “Methods”). **G** Astrocytes showed enriched pathways related to metabolism, gene expression, biosynthesis, and signaling. **H** Microglia showed enriched pathways related to macrophage markers, immune cell development, and cell adhesion. **I** Ependymal cells showed enriched pathways relating to various cellular complexes and binding or response to metal ions.

In astrocytes, a total of 1717 genes were classified as age-associated (Supplementary Data file 6). Similar to other cell types, the majority of these astrocyte aDEGs were unique to a single region, particularly in the EC (*n* = 729; Fig. 4A). Microglia had a total of 676 age-associated genes, and over half were unique to the SVZ (*n* = 344; Fig. 4B and Supplementary Data file 6). Ependymal cells had 479 aDEGs that were limited to the SVZ and EC, and aDEGs unique to the SVZ comprised most of these associations (*n* = 374; Supplementary Data file 6).

Considering the concordance in expression direction between shared aDEGs, each glial cell population exhibits unique regional sharing profiles. Among astrocytes, there was a split between positive and negative instances of concordance (Fig. 4D). Particularly notable instances of sharing included the 40 negatively concordant astrocyte aDEGs shared between the EC and PUT and the positively concordant astrocyte aDEGs shared between the SVZ and PUT (*n* = 21) as well as the SVZ and MTG (*n* = 23; Fig. 4D). In contrast, for microglia, the greatest amount of overlap was between the SVZ and EC for both positively (*n* = 16) and negatively concordant (*n* = 22) aDEGs (Fig. 4E). For ependymal cells, all 10 shared aDEGs were positively concordant and were found in both the SVZ and EC (Fig. 4F). Discordant relationships can be found in Supplementary Fig. 7.

Glial cell aDEG sets yielded 192 enriched pathways with the vast majority being in astrocytes (*n* = 178), particularly within the EC (*n* = 151; Supplementary Data file 8). In the EC astrocytes, the top enriched pathways were linked to metabolic processes, biosynthesis, and regulation of gene expression (Fig. 4G). In the SVZ, astrocyte pathways were more diverse and were linked to cell-cell communication, signal transduction, and response to stimuli (Fig. 4G). PUT astrocyte pathways appear to be specific to the plasma membrane, while MTG astrocyte pathways are involved in cellular maturation and starch/sucrose metabolism (Fig. 4G). In microglia, there were seven total enriched pathways across all regions (Supplementary Data file 8). The most significantly enriched pathway was found in the EC for macrophage markers (Fig. 4H and Supplementary Data file 8). A connection to the immune system was also seen in SVZ microglia, which were enriched for Innate Lymphoid Cell (ILC) family development (WikiPathways: WP3893; Fig. 4H). Cell-cell adhesion was also found as a theme within SVZ and PUT microglia-enriched pathways (Fig. 4H). Ependymal cells had a total of seven enriched pathways, with the most being in the SVZ (Fig. 4I and Supplementary Data file 8). Of these, the most significant was the response to metal ions, while another pathway implicated metallothioneins (Fig. 4I and Supplementary Data file 8).

Prior studies have shown that glial cells can adopt multiple transcriptional states, distinct from cellular identity^25,26^. To determine whether any such glia states were present in our sample series, subclustering was performed on microglia and astrocyte nuclei (see “Methods”). Microglia resolved into five distinct subclusters (Supplementary Fig. 8A, B). Comparing the average expression profiles of each cluster to known myeloid states found across neurodegenerative diseases^27^ suggests the presence of one clear inflammatory state (cluster 2) and the presence of monocytes (cluster 4, Supplementary Fig. 8C). Astrocyte subclustering yielded four subclusters (Supplementary Fig. 8D, E). Interrogation of subcluster average expression profiles to known astrocyte states^27^ indicated a clear distinction between protoplasmic (clusters 1–2) and fibrous astrocytes (cluster 0; Supplementary Fig. 8D). These results indicate that glial cells described here recapitulate previously reported inflammatory signatures in aging, but not disease-associated signatures.

Endothelial and mural cells exhibited relatively few aDEGs (*n* = 47 and *n* = 89, respectively; Supplementary Fig. 9A, B and Supplementary Data file 6), with over half being specific to the entorhinal cortex. Shared aDEGs were rare and limited to pairs of regions (Supplementary Fig. 9C, D), with no discordant or broadly shared genes.

### Comparison to an independent sample series from the prefrontal cortex confirms that aDEGs are likely largely region-specific

There are several recent reports focused on examining the effect of brain aging on gene expression, including a single-nuclei study by Jeffries et al., which used the prefrontal cortex of individuals without neuropathology across the human lifespan. Comparing the Jeffries_PFC_aDEG set with those in our study reveals that, despite a comparable number of donors, our study yielded significantly more aDEGs across nearly all broad cell types (Supplementary Fig. 10A). Including PFC data from this independent sample series supports our interpretation that aDEGs are largely region-specific. For example, in inhibitory neurons, of the 595 aDEGs found in the Jeffries prefrontal cortex dataset, 393 of these are unique to that region (Supplementary Fig. 10B). Moreover, the modest degree of aDEG sharing that we did find was between cortical regions and not shared with subcortical regions. There was a similar pattern of distinctness between regions for OPCs, as of the 263 OPC aDEGs found in the Jeffries study, 215 are unique to that dataset (Supplementary Fig. 10C). Notably, OPCs contain the only instance of an aDEG being shared across all regions and studies (Supplementary Fig. 10C), *UTRN* (Supplementary Data file 9 and 10), which encodes utrophin–a protein involved in linking the extracellular matrix to the actin cytoskeleton that is found in a wide array of tissues^28^.

### Non-pathological aging is not fully explained by the proposed senescence signatures

**Fig. 5:**
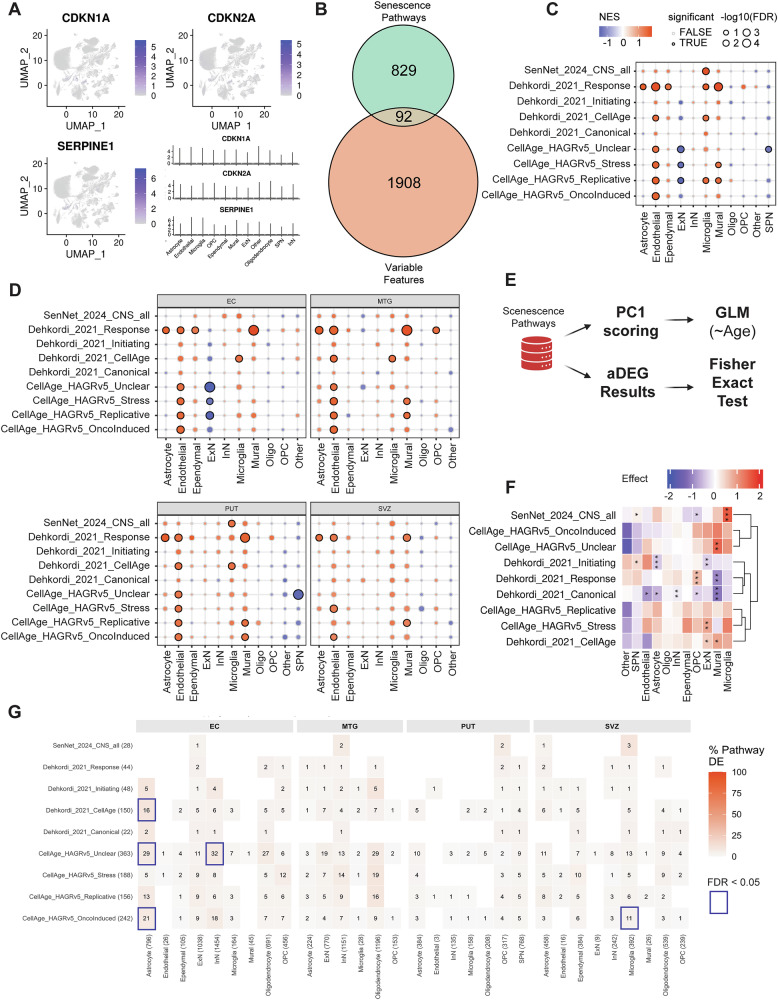
Non-pathological aging is not fully explained by proposed senescence signatures Cellular senescence pathways have been associated with age across tissues^29–31^. **A** Canonical markers of senescence *CDKN1A*, *CDKN2A*, and *SERPINE1* are not highly expressed across cells in this study. **B** 921 gene expression markers of senescence were collected from several independent studies and consortia (Supplementary Data 14), 9.9% of which were present in the variable features defined for clustering. GSEA-based enrichment of senescence pathways across **C** all cell-types in this study and **D** cell-type markers within their respective region (See Supplementary Data 15). **E** Paradigm for testing senescence-associated gene signatures across age. **F** Generalized linear model for the effect of binned age group on per-donor-per-celltype scores of senescence signatures (PC1_signature ~ Age_group; per cell-type across all regions). *FDR < 0.05, **FDR < 0.01, *** FDR < 0.001 corrected across all comparisons (See Supplementary Fig. 11, Supplementary Data 16). **G** Overlap of senescence gene sets (rows, # genes in pathway) and aDEGs per cell-type per region (columns, # aDEG in condition) tested for significance via Fisher’s exact test FDR corrected across comparisons (blue box, FDR < 0.05; See Supplementary Data 17).

There are several recent reports focused on using gene expression changes to infer the presence of cellular senescence^29–31^. Although canonical markers of cellular senescence were not highly expressed across any cell type in our study (Fig. 5A), we wanted to investigate whether senescence-associated gene expression programs were associated with age. A portion of the 921 unique genes found across nine reported signatures of senescence (*n* = 92, Supplementary Data file 14) were present in the variable features used for cluster identification. This result indicates that variation in senescence-linked genes accounts for a subtle proportion of the variation observed in our data. However, gene set enrichment of marker-style differential expression results (Supplementary Data file 15) revealed that these senescence signatures are partially associated with cell identity across (Fig. 5C) and within regions (Fig. 5D). Of note, genes specified as a senescence marker within the central nervous system by the SenNet consortia (“SenNet_2024_CNS_all”)^29^ were strongly associated with microglia identity. Six of the signatures were associated with endothelial and mural cell identity, while two (“Dehkordi_2021_Initiating” and “Dehkordi_2021_Canonical”)^31^ were not associated with any broad cell-type. These patterns were relatively conserved within regions, with the exception of microglia from the putamen, which appear to drive the association to SenNet_2024_CNS_all.

To perform age-association, we asked (1) whether senescence gene programs were associated with age and (2) if our reported aDEGs were enriched for senescence gene signatures (Fig. 5E). We found that the SenNet_2024_CNS_all signature was strongly age-associated in microglia (Fig. 5F and Supplementary Data file 16). Senescence pathways as reported in Dehkordi_2021^31^ appear to show cell-type-specific age-associations across canonical, senescence-responsive, and senescence-initiating sub-pathways. Although the *β*-values from a GLM run on principal components are inherently directionless, no pathway was monotonically associated with age. GLM results on a per-region level exhibited minimal age-association, with only Dehkordi_2021_CellAge and CellAge_HGARv5_Replicative pathways being age-associated in excitatory neurons from the MTG (Supplementary Fig. 11). Finally, over-representation analysis of these senescence gene signatures revealed few significant enrichments in the reported aDEGs (Fig. 5F and Supplementary Data file 17). This finding suggests that the age-associated effects observed in our sample series were not strongly related to cellular senescence. Taken together, these results indicate that senescence pathways may increase in specific cell types with age but are distinct from global age-associated changes in gene expression.

## Discussion

Transcriptomic analysis of postmortem brain samples is integral to the study of age-related diseases; however, little is known about how cell types change during normal aging. Although a limited number of age-matched controls are available across several studies^5,6,32–35^ these are largely collected from the prefrontal cortex. Importantly, it is unknown whether cell types respond to non-pathological aging similarly across regions. There is evidence that the transcriptional state of some cell types, notably astrocytes, is largely regionally defined^36^. However, whether similar age effects can be seen in cell types present in multiple regions has not been robustly investigated. Therefore, there is a need for studies that intentionally target the aging process across multiple brain regions within the same donors. To address these gaps in the literature, we performed single-nucleus RNA sequencing in cohorts of young (20–30 years old) and aged (60–85 years old) individuals across four regions: the entorhinal cortex (EC), middle temporal gyrus (MTG), subventricular zone (SVZ), and putamen (PUT). With this dataset, we found that nearly every cell type, including microglia whose transcriptional states were previously reported not to be defined by brain region^37^, had a region-specific response to age.

Prior bulk RNA sequencing studies on aging reported a decrease in cellular marker gene expression^3,38,39^, but a decrease in gene expression may not necessarily correspond to a physical loss of cells^38,40^. Here, we did not find that age had a significant effect on cell-type proportions across any broad cell-type or region (Fig. 1). Although there is some disagreement in the field regarding whether sampling via single-cell RNA sequencing is an appropriate method for cell counting^18,19^, other studies have reported large cell proportion shifts with age in rodents^41–45^, in animal models of disease^46–53^, and in postmortem human tissues across disease conditions^54–58^. In contrast, we found relatively limited changes in cell numbers. This discrepancy may arise because studies tend to make conclusions about cell loss from data reporting a reduction in cortical volume, which could be suggestive of a reduction in myelination or cell size rather than an overt loss of cells^59,60^. Moreover, stereological counts of specific cell-types are often done in the context of highly-specialized regions, and these studies have shown variability based on the species, the chosen brain region(s), and the cell-types investigated^38,43,61–67^. However, we note that we have a relatively small sample size and that larger studies are needed to draw firm conclusions.

Prior reviews of transcriptional changes with age have emphasized that decreases in synaptic gene expression and increases in microglial activation were common, particularly in the pre-frontal cortex^2^. Consistent with these findings, we have previously reported a decrease in gene expression modules associated with synaptic genes and an increase in modules associated with inflammation^3^. However, most of these claims derive from microarray or bulk RNA-seq data, both of which lack single-cell resolution. By compiling a single nuclei dataset from multiple regions, we describe differentially expressed genes and pathways dysregulated across age on the broad cell-type level for each region. Overall, we report that age-associated differentially expressed genes (aDEGs) tend to be unique to a particular brain region. These aDEGs were mainly protein-coding (Supplementary Fig. 4) and tended to be longer than non-aDEGs (Supplementary Fig. 6). While we found that aDEG expression decreases in inhibitory neurons, inflammatory signals, and other aDEGs tend to be region- and cell-type-specific without much overlap. The cell-type-specific (e.g., microglia compared to astrocytes) as well as region-specific (e.g., SVZ microglia compared to EC microglia) effects reported in our study highlight the importance of atlas-level, multi-region profiling of non-pathological aging.

Our study profiled three broad neuronal cell-types, consisting of inhibitory (InN), excitatory (ExN), and spiny projection neurons (SPN; Fig. 2). We found that inhibitory neurons exhibited the most aDEGs of any neurons, with the largest proportion being in the EC. Of the genes that were shared across regions in inhibitory neurons, the most notable were the 196 aDEGs that decreased in expression with age in both the EC and MTG. Inhibitory neuron aDEGs were functionally enriched for translation and biosynthesis of proteins, particularly in the MTG. Many of the intersecting genes within these pathways encode ribosomal proteins (Supplementary Data file 8). Speculatively, it is possible that impaired ribosomal function leads to dysregulation of translation, potentially resulting in abnormal protein synthesis. These findings align with those of previous studies that have implicated aberrant proteostasis and ribosomal dysfunction in the aging process^21,68–72^. Our study presents the added layer of attributing this dysfunction specifically to inhibitory neurons, which could result in downstream impairments to inhibitory signaling and subsequently disrupt excitatory-inhibitory balance^73–77^. Excitatory neurons showed similar trends to the inhibitory neurons in that most of their aDEGs were specific to the EC, and that the regions with the most pronounced aDEG sharing were the EC and MTG. However, excitatory neurons differed in that they also showed a large number of aDEGs with an age-dependent increase in expression. Given that we observed the presence of region-specific age-effects even in cell-types that are not altered in proportion, our findings lay the groundwork for understanding how different neurons of the brain respond differently to age.

For oligodendrocyte-lineage cells, we found that the aging profiles differed between oligodendrocyte precursors and mature oligodendrocytes (Fig. 3). For example, the MTG had the most region-specific aDEGs for mature oligodendrocytes but the fewest region-specific aDEGs in oligodendrocyte precursor cells. Moreover, both subtypes of oligodendrocyte-lineage cells differed in their functional enrichments. Oligodendrocyte precursor cell aDEGs showed enriched pathways involving neuronal cell components (e.g., membranes, synapse, receptors) while mature oligodendrocyte enriched pathways highlighted post-translational modification, vesicular trafficking/transport, and development. The enrichment of neuronal cell components in the oligodendrocyte precursor cell aDEG sets has been previously reported^78^ and could point towards a dysregulation of OPC-neuronal interactions in aging. OPC-mediated myelination and OPC-neuron synapses have both been shown to decrease with age^79–81^, which could have implications for modulation of neural circuit activity^81,82^. The oligodendrocyte-enriched pathways for vesicular trafficking may also relate to neuronal myelination because this process requires proper lipid delivery mediated by SNARE complex VAMPs^83–85^. In addition, vesicular trafficking has also been shown to regulate oligodendrocyte maturation post-differentiation^86^. Given that our study reported overrepresentation of trafficking processes, this may suggest that oligodendrocyte maturation and myelination are altered during aging, which aligns with previously reported age-related decreases in white matter volume^61,87–89^.

The other glial cell-types represented in our study were astrocytes, microglia, and ependymal cells, and each showed a diverse aging profile (Fig. 4). Astrocytes had the most aDEGs of this subgroup and showed the most significantly enriched pathways of any cell-type, with the largest effect in the EC. These pathways showed clear trends for biosynthesis, gene expression, and several metabolic pathways. Astrocytes use glycolysis for the generation of lactate as an efficient energy source to support the high metabolic demands of neurons^90,91^. This metabolic profile has been reported to shift as a result of aging^92–95^, which could be reflected by changes in relevant gene expression. In contrast to astrocytes, both microglia and ependymal cells showed the most region-specific aDEGs in the SVZ. Of note, these ependymal cell aDEGs were functionally enriched for pathways related to the binding of metal ions. This is consistent with both an emerging role of ependymal cells in metal ion homeostasis^96–98^ and prior descriptions of metal ion handling being disrupted during normative aging and age-related diseases^99–101^. Moreover, there is a growing body of literature suggesting that glia take on plastic, context-specific states during the progression of neurodegenerative diseases^25,26,102^. Our analysis failed to identify disease-associated microglia^27,103–105^ or disease-associated astrocyte states in normative aging (Supplementary Fig. 8). Future studies performing glial-enrichment prior to single-cell profiling of an aging cohort will be necessary to characterize the presence of glial substates more fully.

Taken together, our differential expression findings support that cell types have discrete, non-overlapping aging signatures across regions. This presents a challenge for the generalizability or discovery of driver genes of aging. Recent work on calculating biological age from algorithms (frequently called clocks) has revolutionized the field of gerontology^106,107^. Many of these biological clocks are trained on bulk tissue and meant to generalize across studies^108–110^. However, the performance of such clocks in single-cell data remains poorly documented. There has been some effort to create cell type-specific clocks^111^, to do so requires source- and species-specific observations to be regressed out. Given our findings that transcriptomic signatures are region-specific, future RNA age clocks or other models for age prediction ought to consider that cell types may age differently according to their region of origin. As more multi-regional atlases are collected, future studies will be required to identify the extent to which region impacts cell type-specific responses to age.

Notably, our study is only the third single-nuclei study of the adult SVZ. Previous studies focused either on aged individuals alone^112^ or the comparison between the young and middle aged^113^. Our finding of large age-related shifts in glia is consistent with these two previous studies, the region remains understudied despite its significant role in development^114,115^ and disease^38,116–120^. This is especially critical as the SVZ would be the site of adult neurogenesis^17,121–123^, and there is evidence suggesting that impaired neural stem cell function may be implicated in neurodegenerative disorders^124–126^. The largest of the two previous studies compared age groups of 16–22 (young) and 44–53 (middle-aged) with particular attention to developmental processes^113^. They found that while neural stem cell numbers did not decline across their sample ages, oligodendrocyte precursor cells and microglia did, along with an associated decrease in developmental genes. The authors suggested that these findings pointed to a remodeling of the SVZ between youth and adulthood. While our study is similarly powered in terms of donors and cells captured, the disparity in findings may relate to the specific chosen age range in the older group (Puvogel et al.: 44–53 years versus the current study: 60–85 years). As more SVZ samples become available and single-nuclei data are collected, future studies will be necessary to separate periods of development from any eventual decline with age.

Several studies have highlighted the role of cellular senescence in the aging process^29,31,127^. While senescence-associated genes made only a modest contribution to the global variation in our dataset, we found that several curated senescence pathways are significantly enriched for cluster markers. This result suggests that despite being curated for senescence, these gene sets overlap with genes that partially define cellular identity. Previous work on fibroblasts and endothelial cells has similarly shown that these senescence gene lists need to be further refined to accurately distinguish functional senescence^128^. However, testing these senescence signatures within cell types reveals sparse associations with age (Fig. 5). While the eigengenes of some senescence pathways were significantly associated with age, these findings were almost exclusively seen at the broad cell type level. Furthermore, the age-associations we report in this study (aDEGs) are not largely explained by senescence pathways as they are presently annotated. Taken together, these findings suggest that cellular senescence does not fully explain all aspects of non-pathological brain aging. To assess whether a causal relationship between aging and senescence exists, future studies should pair region- and cell type-specific gene expression to functional measures of cellular senescence.

Our sample series prioritizes large effect sizes between young (20–30 years old) and aged (60–85 years old) cohorts and represents the largest multi-region, non-pathological brain aging study to date (*n* = 5–7 donors × 4 regions). One limitation of the current design is a lack of middle-aged samples, limiting our ability to identify gene expression changes along continuous chronological age. Future studies with continuous age sampling would allow the identification of novel aging trajectories for which the current study is not designed. Additionally, our findings are all based on differential gene expression. Although sequencing-based transcriptomic technologies are the most routinely used methods to profile unbiased changes at single-cell resolution, profiling at the epigenetic and proteomic levels is not captured by the current study. As single-cell and spatial technologies expand to include other readouts such as global proteomics, DNA accessibility, and nucleotide modifications, these additional modalities have the potential to enhance our understanding of how gene-regulation changes with age. Lastly, while the non-pathological design can be applied to the idea of healthy or resilient aging, future work should focus on the comparison to age-related diseases such as Alzheimer’s and related dementias.

## Methods

### Brain bank information and sample selection

Brain tissue samples were obtained from the National Institute of Mental Health (NIMH)’s Human Brain Collection Core (HBCC). Brain donors were identified through the Office of the Chief Medical Examiner (OCME) of the District of Columbia and the Northern District of Virginia. Consent for use of tissue samples was provided by each donor’s next-of-kin. Use of postmortem human brain tissue samples is not considered human subjects research under current federal regulations, so IRB review and approval were not required. Since the present study intended to investigate the molecular mechanisms of normal aging, we solicited tissue samples from individuals who had *not* been diagnosed with any neuropsychiatric disorder in their lifetime. In lieu of quantified neuropathology scores (i.e., Braak Tau, Braak Syn, Thal), selected samples did not have apparent evidence for neurodegeneration. Pathological assessment was performed by a neuropathologist. Procedures for assessment and characterization of cases are described in previous publications^129^.

Suitable donors had usable tissue across the four brain regions of interest: the entorhinal cortex (EC), middle temporal gyrus (MTG), subventricular zone (SVZ), and putamen (PUT). We selected individuals with the lowest available postmortem interval (PMI range: 13–58 h.) and brain pH levels (range: 6.19–6.92). Samples were classified by age, with young being 20–30 years old (*n* = 6; 3 per sex) and aged being 60–85 years old (*n* = 7; 4 male, 3 female) at the time of death. Due to tissue availability or sample processing failure, three donors are not full-rank. However, each region has contributions from 12 independent donors (5–7 per age condition; see Supplementary Fig. 1 and Supplementary Data file 1 for sample characteristics and demographic info.).

### Isolation of nuclei from the human brain

Nuclei were isolated using the Nuclei PURE Prep Nuclei Isolation Kit (Sigma #NUC201) per the manufacturer’s instructions. To streamline the workflow and minimize potential batch effects, we utilized a pooling approach prior to library preparation and proceeded with post-sequencing demultiplexing, generating six pools of eight samples each. Each pool was balanced for age group and sex, ensuring that no single pool contained two regions from the same donor.

A 100 mg piece of tissue was placed on a fresh, pre-chilled petri dish, trimmed, and weighed. Within each of the six pools, approximately 25 mg of tissue for each of the eight samples was combined and homogenized in a single cold douncer containing 2 mL ice-cold lysis buffer, Nuclei PURE Lysis Buffer (Sigma #L9286), 0.1 M freshly thawed DTT (Sigma #GE17-1318-01), and 0.1% Triton X-100 (Thermofisher Scientific #T1565). Tissue was homogenized with 25 strokes of a loose pestle followed by 25 strokes of a tight pestle, transferred to a tube containing 8 mL cold lysis buffer, vortexed 2–3 s, and left on ice for 10 min. Following lysis, cold 1.8 M sucrose cushion solution (Nuclei PURE 2 M Sucrose Cushion Solution (Sigma #S9308), Nuclei PURE Sucrose Cushion Buffer (Sigma #S9058), and 0.1 M DTT) was added to the bottom of an ultracentrifuge tube (Beckman Coulter #344058) on ice. To each lysate, cold 1.8 M sucrose cushion solution was added and mixed using a serological pipette. The lysate solution was slowly layered on top of the sucrose cushion, placed in a pre-cooled ultracentrifuge, then centrifuged for 45 min at 30,000 × *g* at 4 °C.

Sample tubes were removed from the ultracentrifuge and placed on ice. The entirety of the supernatant was aspirated, and the nuclei pellet was resuspended in Nuclei Suspension Buffer on ice (NSB; 1 mL cold PBS) (Thermofisher Scientific #10010-023), 0.01% BSA (New England Biolabs #B9000S), 0.1% SUPERase RNase inhibitor (Thermofisher Scientific #AM2696), transferred to a 15 mL tube containing an additional 4 mL NSB buffer, mixed, and washed by centrifugation at 500 × *g* for 5 min at 4 °C. The pellet was resuspended in 1 mL NSB, filtered through a 70 μM Cell Strainer (STEMCELL Technologies #27216) to remove debris, and washed again via centrifugation. The supernatant was aspirated, and nuclei were resuspended in a final volume of 110 μL.

To determine the nuclei concentration, Acridine Orange/Propidium Iodide (Logos Biosystems #F23001) was added to the nuclei suspension in a separate tube and counted using a LUNA-FL Dual Fluorescence Cell Counter (Logos Biosystems). An appropriate volume of nuclei was diluted with NSB to achieve a final single suspension of 80,000 nuclei (per sample) to maximize nuclei recovery while minimizing the multiplet rate. Approximately 10,000 nuclei from this single suspension were loaded into each of 8 lanes of a 10x Genomics NextGEM Chip G (10x Genomics #1000127) and inserted into a 10xGenomics Chromium Controller (10xGenomics #1000204) according to the manufacturer’s instructions. We loaded 10 K nuclei per lane, targeting ~6 K nuclei recovered per lane with a multiplet rate of ≤5%. The remainder of library preparation was conducted according to the Chromium Single Cell 3’ Reagent Kits User Guide (v3.1 Chemistry; Rev.D). Final libraries were sequenced at the NIH Intramural Sequencing Center (NISC) at a read depth of 25,000 paired-end reads per nucleus on an Illumina NovaSeq 6000. Data was processed with CellRanger count v5.0.1 with refdata-gex-GRCh38-2020-A as the reference with introns included.

### Genotype preparation and demultiplexing of pooled samples

Genotypes were assayed from three array-based genotyping platforms: Human1M-Duov3_B, HumanHap650Yv3.0, and HumanOmni5-Quad. Genotypes were merged and filtered to variants that were common to all three platforms, called in 95% of the samples, and known to be single-nucleotide variants (SNVs). Genotype files were lifted over from hg19 to hg38. The Plink2.0^130^, bcftools^131^(version 1.12), and Picard (version 2.25.5) (https://broadinstitute.github.io/picard/) tool sets were used to filter, format, and lift over the genotype files. Pooled samples were demultiplexed using the demuxlet tool (version 2)^132^. The prepared subject genotypes and the aligned single-nuclei bam files were used to deconvolute the cells’ sample identities. Data were processed on the Google Cloud Platform (GCP) using the Cumulus/Demuxlet workflow^133^ (WDL, https://cumulus-doc.readthedocs.io/en/0.12.0/demuxlet.html), which executes the demuxlet tool contained in the Statgen Popscle suite (https://github.com/statgen/popscle). Job submission to GCP for execution was done via the Broad WDL runner (https://github.com/broadinstitute/wdl-runner) and GCP Life Sciences interface (https://cloud.google.com/life-sciences/docs). SCANPY^134^ (version 1.7.1) was used to read in the 10X filtered matrix files into an AnnData object and integrate sample identity for the deconvoluted cells. Cells that were scored with ambiguous genotype calls (22.3% of sequenced nuclei; per pool mean 21.6% ± 6.5%) or called as heterogenic doublets (10.7% of sequenced nuclei; per pool mean 0.1% ± 2.3%) were excluded. Nuclei assigned to donors outside of the corresponding pool were also excluded (0.3% of sequenced nuclei; per pool mean 0.3% ± 0.1%). For all demultiplexing information, see Supplementary Data file 11.

### Clustering and cell-type identification

The single-cell analysis tool Pegasus^133^ (version 1.3) (https://pegasus.readthedocs.io/en/stable/index.html) was used to combine, filter, normalize, cluster, and determine the initial cell-type identities of the demultiplexed single-nuclei count data. Basic filtering was done with Pegasus, excluding cells that did not include at least 200 genes, genes that were not present in at least three cells, and cells that had a mitochondrial DNA content of over 10%. Clustering was performed on log-normalized counts using the top 2000 variable genes. Harmony-corrected principal components were calculated for integration across samples and assessed for quality control with scIB^135^; Supplementary Fig. 2A. Clusters were identified using fast approximate nearest neighbor search (hnswlib^136^). The Leiden^137^ algorithm (version 0.8.3) was used to identify clusters on the neighborhood graph, and multiple resolutions were inspected to determine appropriate cell-type identification.

Initial inferences for the putative cell-type identity of each cluster were made based on a combination of the computed marker genes for that cluster compared to all others and the relative expression levels of canonical marker genes for different CNS broad cell-types (For a list of the marker genes, calculated with default settings in Pegasus, see Supplementary Data files 3, 4, and Supplementary Fig. 2). To preserve the number of expected cell-types in a single round of clustering, we selected a final Leiden resolution of 0.85. Cellbender^138^ was run to determine the degree to which ambient RNA may impact the cell data and clusters. Of the cells detected as ambient by Cellbender, 98.9% of these cells were filtered out by either CellRanger or demuxlet. For those filtered by demuxlet, the majority were labeled as ambiguous. Of the small fraction of remaining ambient cells, 76.6% of these were assigned to a cluster within the “Other” cell-type, where 21.3% of cells within the “Other” clusters were scored as ambient. In cases where a broad-cell-type annotation could not be definitively assigned via algorithmic or manual inspection or were determined to be biologically inappropriate (e.g., cells labeled as “Spiny Projection Neurons” but were localized to regions other than the Putamen), these cells were labeled as “Other” and excluded from further analysis (*n* = 16,298 nuclei). As a separate analysis, sub-clustering of SPNs performed in Seurat^139^ and integrated with Harmony^136^ further demonstrates that dopaminergic neurons in this study are largely contributed by the putamen (Supplementary Fig. 3). The final accepted dataset consisted of 151,647 nuclei, representing 10 annotated cell-types.

### Cell-type proportion and regional specificity analyses

In order to compare cell type proportions across age, nuclei counts by region per age group were obtained and this value was used to calculate the percentage of nuclei that this cell type comprised within that particular region and age group (see values in Supplementary Data file 2; percentages in Fig. 1F). To evaluate whether these differences in nuclei numbers between age groups were due to a significant effect of age on cell-type proportions, the *propeller*^140^ function of the R-Package *speckle* (version 1.60) was used with default parameters as recommended by the authors. In brief, this function applies a logit transformation to per-sample cell counts followed by a t-test to compare the age effect (young vs. aged) in each cell-type. This method was applied within each region and adjusted the *p*-values using the Benjamini and Hochberg False Discovery Rate (FDR-BH) method prior to determining significance for all comparisons across the 4 regions. For ease of interpretation, summary proportions per cluster were then normalized (−1: 100% young; 1: 100% aged).

To assess whether any cell types were more specific to a particular region, a regional specificity value was calculated for each cell-type by region subgroup. These specificity values were calculated by determining the total number of cells of a given type within the region of interest and dividing that by the total number of cells of that type across all regions (e.g., # ExN_MTG / # ExN_total). For example, if a cell-type-region subgroup had a specificity value of 0.5, 50% of all cells of that type were contained within that one region. Source code for the determination and heatmap visualization of both proportion and regional specificity values can be found on GitHub.

### Age-related differential expression and association

Differential expression analysis was performed with age group as the independent variable per broad cell-type per region. Age was treated as a binary, categorical variable given as “young” (20–30 years) or “aged” (60–85 years). To exclude poorly mapped genes, we applied a filter requiring that genes tested for differential expression must have at least one read in a minimum of 3 cells for at least 50% of subjects. For computational efficiency, the differential expression analysis was performed in two steps.

First, a simple t-test between age groups was performed using the *diffxpy* package (https://diffxpy.readthedocs.io/en/latest/index.html). Any result exhibiting a nominally significant difference was considered to proceed to the second step. In this second step, to address the impacts of pseudoreplication and zero-inflation, we utilized a generalized linear mixed model (GLMM) with a Tweedie distribution^141^. Specifically, to account for pseudoreplication, a random-effect term was included to account for the sample, while a Tweedie distribution was specified to account for zero-inflation. Additionally, the pool number was included as a covariate term to account for residual batch effects that were not corrected with Harmony. The glmmTMB^142^ R package was used to run this model: gene ~ age_group + pool + (1|sample_id). To correct for multiple testing across both cell-type and region, the resulting p-values were adjusted using the Benjamini and Hochberg False Discovery Rate (FDR-BH) method, as implemented in the *statsmodel multitest* Python package (https://www.statsmodels.org). In order to evaluate genes that were differentially associated with age (aDEGs) in a particular cell-type, the Python package *UpSetPlot* (version 0.9.0) (https://upsetplot.readthedocs.io/en/stable/#) was used to visualize aDEG distribution and sharing across regions. All UpSet Plots are sorted by cardinality with no cutoff parameters. The input dataframes for each UpsetPlot can be found in Supplementary Data file 12.

### Evaluating concordance/discordance of adeg expression direction across regions

Given that there were aDEGs shared across two or more brain regions within a given cell-type, we investigated whether the expression of these genes changed in the same or opposing directions with age between pairs of regions of interest. A concordant signature had the aDEG increasing (up/up) or decreasing (down/down) with age in both regions. A discordant signature had the aDEG increasing in one region and decreasing in another (up/down or down/up). The input dataframes for each heatmap can be found in Supplementary Data file 13.

### Functional enrichment analysis with background correction

Functional enrichment analysis was performed on each of the aDEG sets determined by the custom generalized linear mixed model (FDR BH adj. *p* < 0.05) within each broad cell-type, brain region subgroup using the R package *gprofiler2*^143^ (version 0.2.3). For each cell-type by region aDEG set, all genes were evaluated together, independent of effect direction (i.e., genes increasing in expression with age were not evaluated separately from those decreasing) in order to preserve biological context. In addition, all reference gene sets for tested pathways were background-corrected for genes that were generally expressed (i.e., not an aDEG) in a given cell-type by region subset (i.e., they were removed from the reference set).

### Comparison to previously published signatures and findings

In order to compare findings in the present study to those previously published, we implemented a multi-faceted strategy, including gene-set comparison and cluster similarity analysis implemented in SAHA^144^. First, for gene set comparisons, summary statistics for differentially expressed genes associated with age were downloaded as previously reported^7^. Per-region per-cell type aDEGs, as reported in this study, were compared to per-cell type elderly_vs_adult (adult: 15–57; *n* = 7 vs. elderly 66–104; *n* = 10) results, all within the prefrontal cortex. Second, to test the existence of glial cell substates across our non-pathological aging cohort, we subclustered cells annotated as microglia (*n* = 3265) and astrocytes (*n* = 11,289) in Seurat^139^ and integrated across donors with Harmony^136^. K-nearest neighbor clustering (astrocytes, n_PCs = 3; microglia, n_PCs = 3) followed by cluster discovery (astrocytes, resolution = 0.1; microglia, resolution = 0.1) revealed 4 astrocyte and 5 microglia subclusters. Average expression profiles were calculated in SAHA^144^ and compared to average expression profiles from literature^27^.

To investigate whether the present study contains evidence for age-related senescence across cell types or regions, we curated a list of non-redundant senescence signatures. These include SenNet (National Institutes of Health (NIH) Cellular Senescence Network SenNet Consortium)^29^, CellAge (Human Ageing Genomic Resources [HAGR] Database of Cell Senescence Genes)^30^, and divergent senescence sub-pathways observed in human neurons^31^. All possible signatures found in these resources were investigated, along with markers from two separate studies^127,128^. However, as multiple lists were highly redundant, we selected the 9 signatures that were divergent in either genes listed or size of pathway (Supplementary Data File 14). For consistent naming, we label each by source or last author (i.e., “CellAge_”) followed by year or version (i.e., “HAGRv5_”) and finally the name from the original source (i.e., “Unclear” for HAGR-annotated senescence-associated genes or unknown involvement). Specifically, for the expert recommendations from SenNet, we filtered for genes marked as “CNS”, but found that further filtering for those markers labeled “scRNA-seq” did not appreciably change the list. As a result, we decided to test the entirety of the SenNet CNS-flagged signature (hence the name “SenNet_2024_CNS_all”). Because these signatures are not explicitly proven to be age-associated, we employed three different analyses. First, we performed a GSEA-based enrichment for senescence pathways against marker gene results (Seurat::FindAllMarkers(…, logfc.threshold = 0, min.pct = 0, only.pos = FALSE)) in the integrated dataset and within each region (Supplementary Data File 15). Second, we scored each pathway on a per cell-type per region per donor basis using the first principal component of normalized read counts for the genes in each pathway. Following PC1 calculation, we ran a GLM against age group (Young vs. Aged, Supplementary Data file 16). Third, we performed a Fisher’s exact test between curated senescence pathways and the aDEGs present in our study. *P*-values for each of the three tests were FDR-corrected across all comparisons (Supplementary Data file 17).

### Online supplemental material

Supplemental tables include: a donor- and cell-level metadata (Supplementary Data files 1, 2), cluster markers and cell taxonomy (Supplementary Data files 3–5), aDEG summary information and full enrichment results (Supplementary Data files 6-8), cross-study analysis summary (Supplementary Data files 9, 10), genetic demultiplexing results (Supplementary Data file 11), age-effect directionality information (Supplementary Data files 12, 13), and investigation of cellular senescence (Supplementary Data files 14–17).

## Supplementary information

Mesecar_SupplementaryFiguresLegends_Combined_UPDATED_March2026

SupplementaryData1_DonorInfo

SupplementaryData2_NucleiCount

SupplementaryData3_leiden085_markers

SupplementaryData4_broad_CT_markers

SupplementaryData5_CellTaxonomy

SupplementaryData6_aDEGs

SupplementaryData7_wilcoxon_gene_length_summary

SupplementaryData8_SignifEnrich_BckgrCorr

SupplementaryData9_Jeffries_Summary_DEGoverlap

SupplementaryData10_Jeffries_PerCellType_DEGoverlap

SupplementaryData11_Demultiplexing_Metrics_Info

SupplementaryData12_UpSet_Input

SupplementaryData13_ConDiscordance_Input

SupplementaryData14_SenescencePathways

SupplementaryData15_FGSEA_Markers

SupplementaryData16_PCA_GLM

SupplementaryData17_aDEG_Senescence

## Data Availability

Raw single-nucleus RNA sequencing data are available in the NIMH Data Archive associated with the collection “Human Brain Collection Core genomics data in postmortem brain of psychiatric disorders #3151” (https://nda.nih.gov/edit_collection.html?id=3151); experiment ID 2370: “snRNA_brain_aging.” De-identified individual level meta-data used as either selection criteria and/or covariates in analysis can be found in Supplementary Data file 1. Summary-level results and processed single-cell objects are available on Zenodo (10.5281/zenodo.7803697) and in Supplementary Data files 8, 12-13. Associated code used for analysis and plot generation are available on GitHub (https://github.com/neurogenetics/ADRD_Brain_Aging/tree/main/phase1).
