# Supplementary material for: Region-specific transcriptional signatures of brain aging in the absence of neuropathology at the single-cell level
Source: NPJ Aging. 2026 May 13;12(1):100. doi: 10.1038/s41514-026-00391-9 (PMC13396485; doi:10.1038/s41514-026-00391-9)
Supplement: Supplementary file 1 — Mesecar_SupplementaryFiguresLegends_Combined_UPDATED_March2026 [file 41514_2026_391_MOESM1_ESM.pdf]

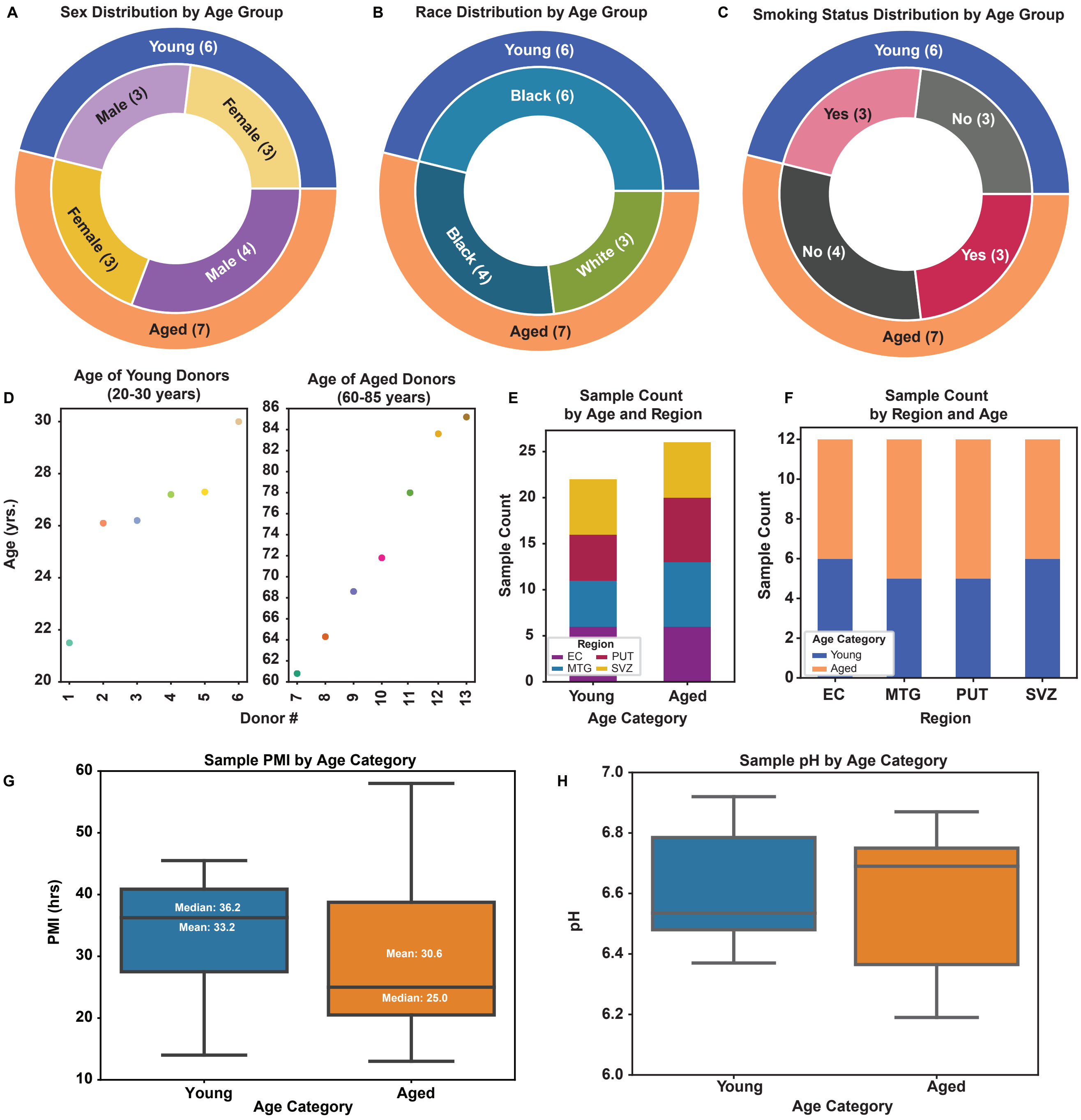
A
Sex Distribution by Age Group
Young (6)
Male (3)
Female (3)
Female (3)
Male (4)
Aged (7)
B
Race Distribution by Age Group
Young (6)
Black (6)
Black (4)
White (3)
Aged (7)
C
Smoking Status Distribution by Age Group
Young (6)
Yes (3)
No (3)
No (4)
Yes (3)
Aged (7)
D
Age of Young Donors (20-30 years)
Age of Aged Donors (60-85 years)
Age (yrs.)
Donor #
E
Sample Count by Age and Region
Sample Count
Region
EC
PUT
MTG
SVZ
Young
Aged
Age Category
F
Sample Count by Region and Age
Sample Count
Age Category
Young
Aged
EC
MTG
PUT
SVZ
Region
G
Sample PMI by Age Category
PMI (hrs)
Median: 36.2
Mean: 33.2
Mean: 30.6
Median: 25.0
Young
Aged
Age Category
H
Sample pH by Age Category
pH
Young
Aged
Age Category

Supplementary Figure 1: Donor Demographics and Sample Characteristics

Distribution of demographic characteristics across 13 donors within young (20-30 yrs.) versus aged (60-85 yrs.) groups: **(a)** sex, **(b)** race, **(c)** smoking status, and **(d)** age. Distribution of tissue sample counts **(e)** by region within an age category and **(f)** by age category within a region of interest demonstrates a relatively even sample distribution. This sample series was selected based on minimizing variability in **(g)** post-mortem interval (PMI; range 13-58 hrs; mean: 33.2 hrs.; median: 36.2 hrs.) and **(h)** brain pH (range 6.19-6.92).

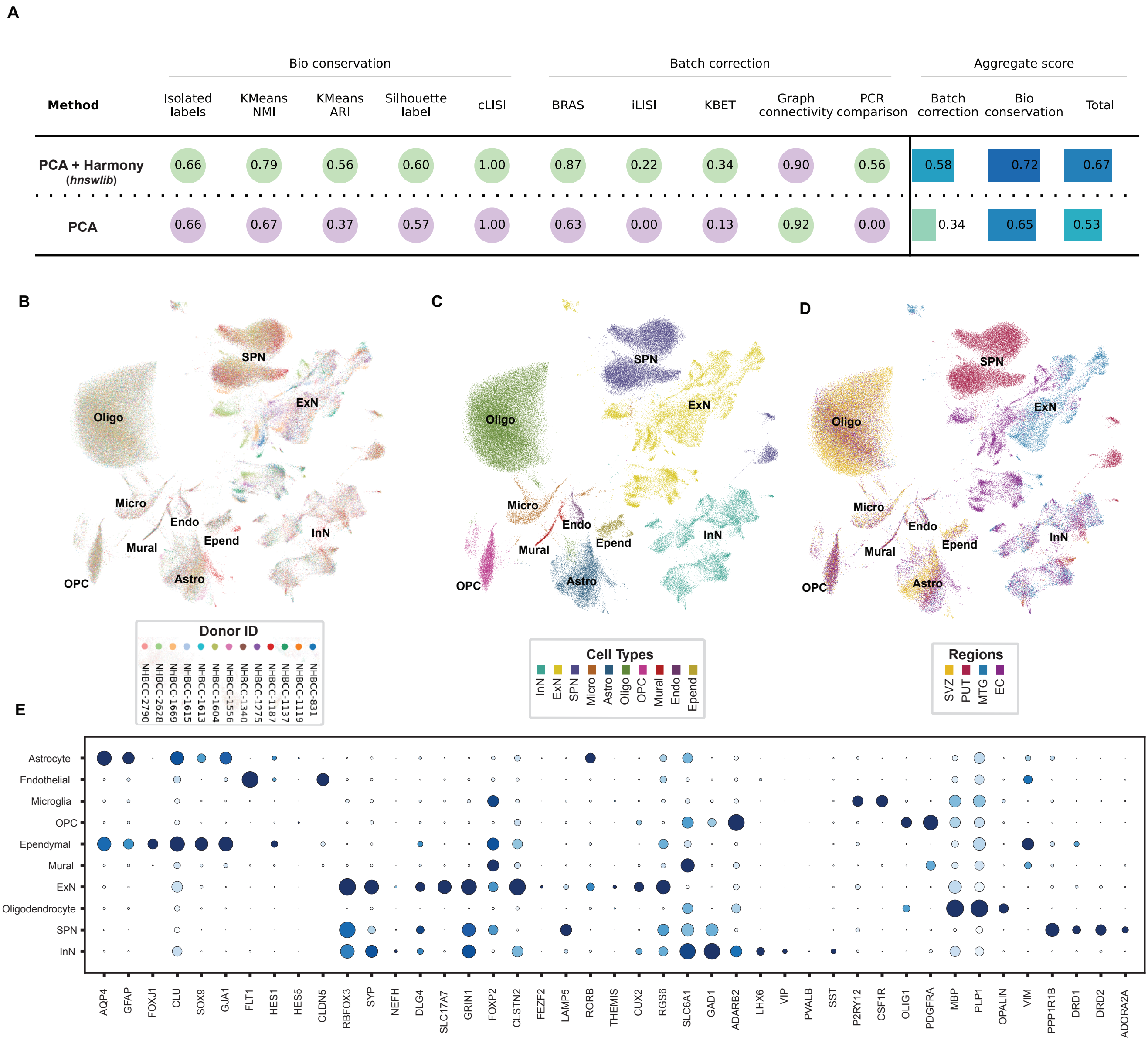
A
Bio conservation
Batch correction
Aggregate score
Method
Isolated labels
KMeans NMI
KMeans ARI
Silhouette label
cLISI
BRAS
iLISI
KBET
Graph connectivity
PCR comparison
Batch correction
Bio conservation
Total
PCA + Harmony (hnswlib)
0.66 0.79 0.56 0.60 1.00 0.87 0.22 0.34 0.90 0.56 0.58 0.72 0.67
PCA
0.66 0.67 0.37 0.57 1.00 0.63 0.00 0.13 0.92 0.00 0.34 0.65 0.53
B
C
D
SPN
ExN
Oligo
Micro
Endo
Mural
Epend
InN
OPC
Astro
Donor ID
NHBCC-2790 NHBCC-2628 NHBCC-1669 NHBCC-1615 NHBCC-1613 NHBCC-1604 NHBCC-1556 NHBCC-1340 NHBCC-1275 NHBCC-1187 NHBCC-1137 NHBCC-1119 NHBCC-831
Cell Types
InN ExN SPN Micro Astro Oligo OPC Mural Endo Epend
Regions
SVZ PUT MTG EC
E
Astrocyte
Endothelial
Microglia
OPC
Ependymal
Mural
ExN
Oligodendrocyte
SPN
InN
AQP4 GFAP FOXJ1 CLU SOX9 GJA1 FLT1 HES1 HES5 CLDN5 RBFOX3 SYP NEFH DLG4 SLC17A7 GRIN1 FOXP2 CLSTN2 FEZF2 LAMP5 RORB THEMIS CUX2 RGS6 SLC6A1 GAD1 ADARB2 LHX6 VIP PVALB SST P2RY12 CSF1R OLIG1 PDGFRA MBP PLP1 OPALIN VIM PPP1R1B DRD1 DRD2 ADORA2A

Supplementary Figure 2: Harmony-corrected PCA yields improved integration across 25 Leiden clusters representing 10 unique broad cell types from four brain regions.
(a) Harmony-corrected PCA offers improved batch-correction metrics (scIB[130]) over PCA alone. (b) Leiden clustering of 151,647 nuclei at 0.85 resolution resulted in 25 distinct clusters, labeled by cell-type (n=10). Coloring by donor reveals heterogeneous clusters, indicating successful integration. (c) Leiden clusters re-colored according to broad cell-type annotation and (d) brain region of origin. (e) Relative expression levels and proportion of cell-types expressing canonical marker genes were used to manually annotate to the broad cell-type level.

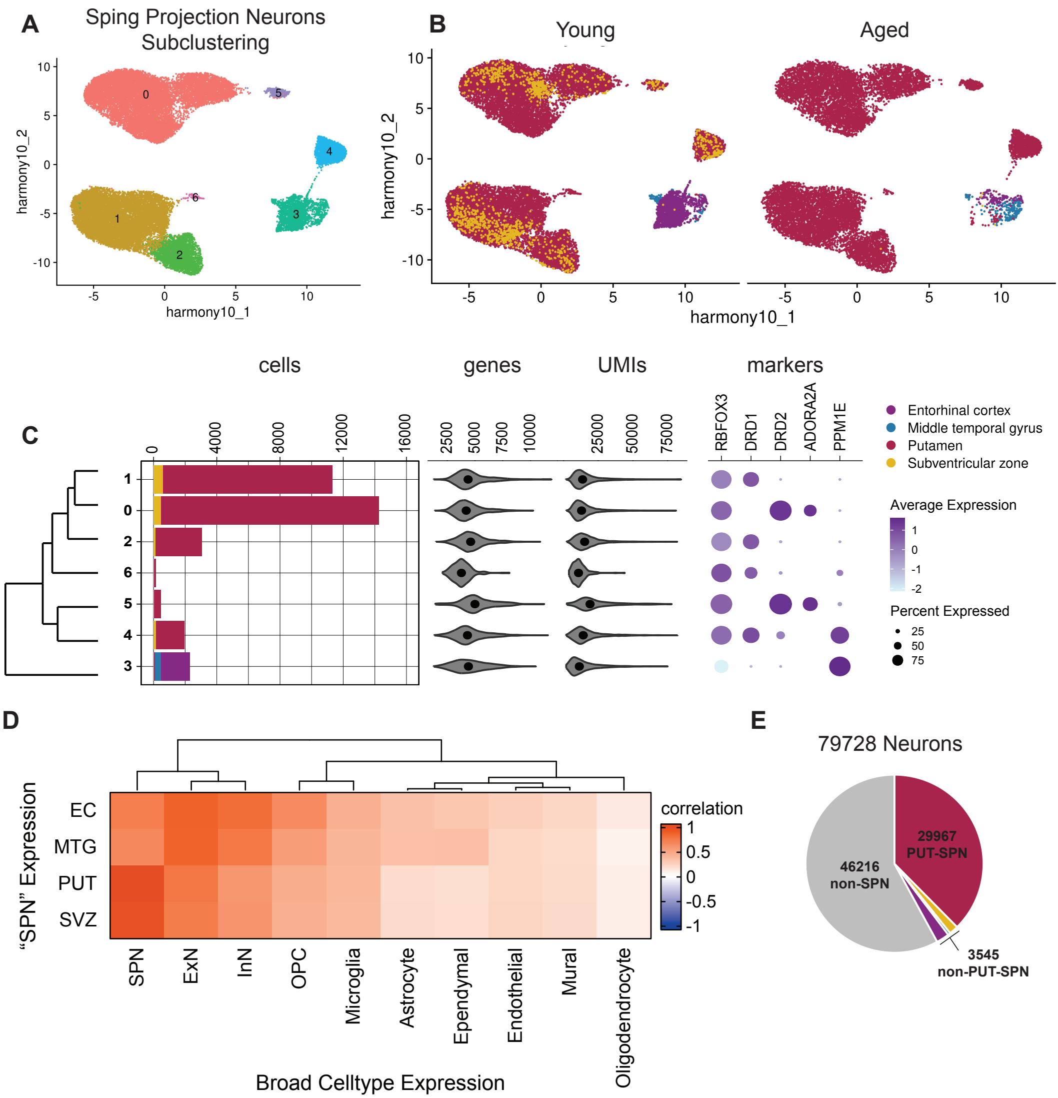
A
Sping Projection Neurons
Subclustering
harmony10_2
harmony10_1
B
Young
Aged
harmony10_2
harmony10_1
C
cells
genes
UMIs
markers
RBFOX3
DRD1
DRD2
ADORA2A
PPM1E
Entorhinal cortex
Middle temporal gyrus
Putamen
Subventricular zone
Average Expression
Percent Expressed
D
"SPN" Expression
EC
MTG
PUT
SVZ
correlation
SPN
ExN
InN
OPC
Microglia
Astrocyte
Ependymal
Endothelial
Mural
Oligodendrocyte
Broad Celltype Expression
E
79728 Neurons
29967
PUT-SPN
46216
non-SPN
3545
non-PUT-SPN

Supplementary Figure 3: Subclustering of nuclei annotated as spiny projection neurons affirms regional specificity for putamen and indicates probable misannotated nuclei
(a) Subclustering of nuclei annotated as spiny projection neurons in the initial round of clustering results in 7 subclusters. **(b)** Separating these nuclei by age group (left: young; right: aged) and coloring them by brain region of origin reveals that subclusters (0-2 and 5-6) largely come from the putamen. Nuclei from other regions were few in number (n=3,545), so they were re-annotated as "Other" and excluded from further analysis. (**c**) Quality control summary statistics at the subcluster level include cell counts per region, unique genes expressed, total counts, and relative normalized expression levels for known SPN marker genes. (**d**) Average expression profile comparison between annotated SPNs per region and broad cell types performed in SAHA[139]. Spearman correlation of the normalized expression of top 2,000 variable features calculated in Seurat[134] reveal MTG- and EC-derived SPNs more closely match ExN and InN profiles than SPNs together (largely PUT-derived). (**e**) Distribution of 79,728 neurons in this study (ExN, InN, SPN) demonstrate that SPNs outside of the PUT make up 4.45% of total post-QC neurons, while PUT-derived SPNs comprise 37.59% total of total post-QC neurons.

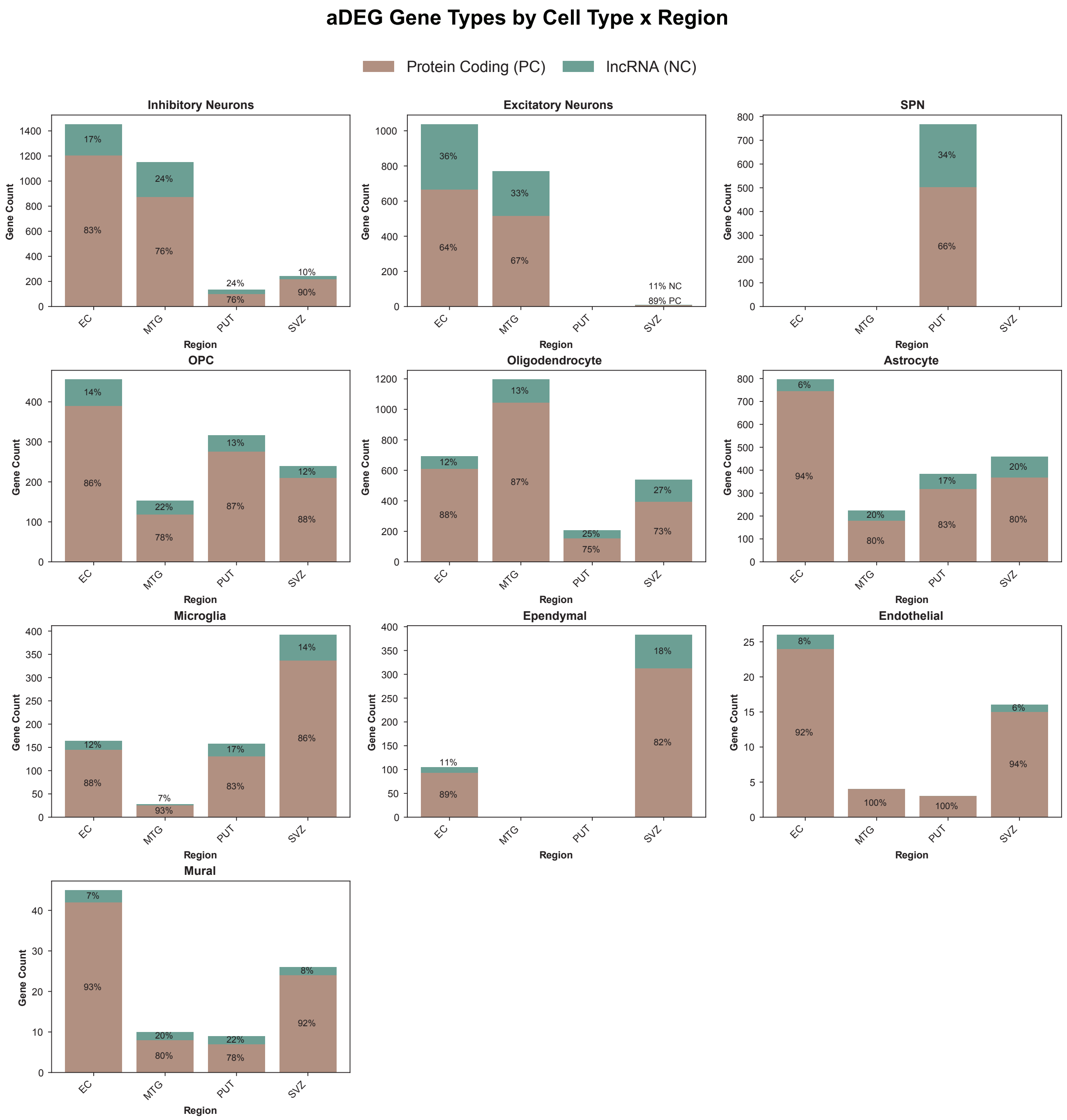

aDEG Gene Types by Cell Type x Region
Protein Coding (PC)
lncRNA (NC)
Inhibitory Neurons
Excitatory Neurons
SPN
OPC
Oligodendrocyte
Astrocyte
Microglia
Ependymal
Endothelial
Mural
Gene Count
Region
EC
MTG
PUT
SVZ
11% NC
89% PC

Supplementary Figure 4: Majority of aDEGs are protein coding across cell-types and regions

Classification of aDEGs as protein coding or long non-coding RNAs (annotations associated with refdata-gex-GRCh38-2020-A) across all cell-type-region subgroups reveals that the majority of aDEGs are protein coding.

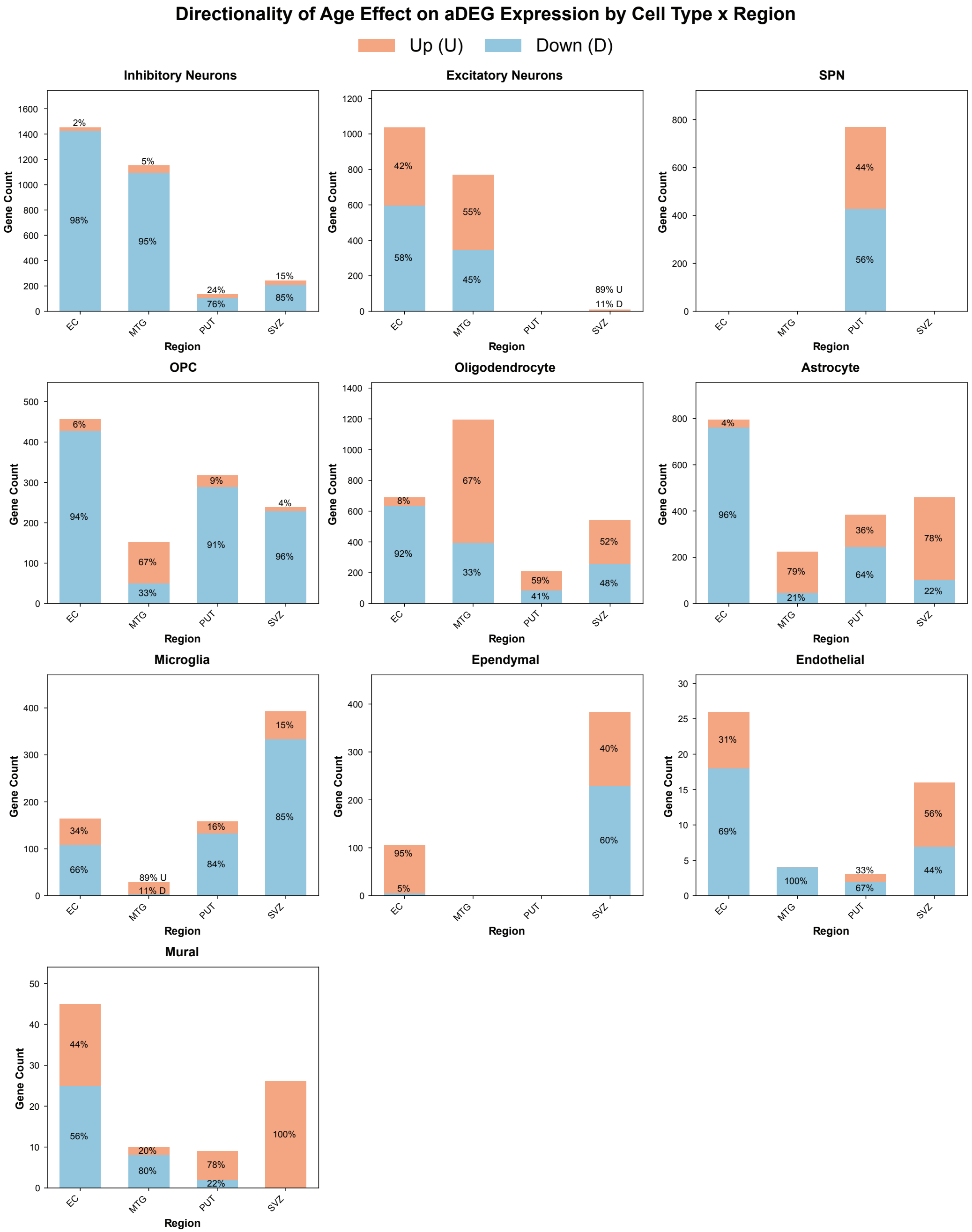

Directionality of Age Effect on aDEG Expression by Cell Type x Region
Up (U)
Down (D)
Inhibitory Neurons
Excitatory Neurons
SPN
OPC
Oligodendrocyte
Astrocyte
Microglia
Ependymal
Endothelial
Mural
Gene Count
Region
EC
MTG
PUT
SVZ
89% U
11% D

Supplementary Figure 5: aDEG expression levels tend to decrease with age across most cell-type by region subgroups

aDEG expression direction (i.e. increase or decrease) across all cell-type-region subgroups suggests that overall, the majority of aDEGs tend to decrease in expression with age.

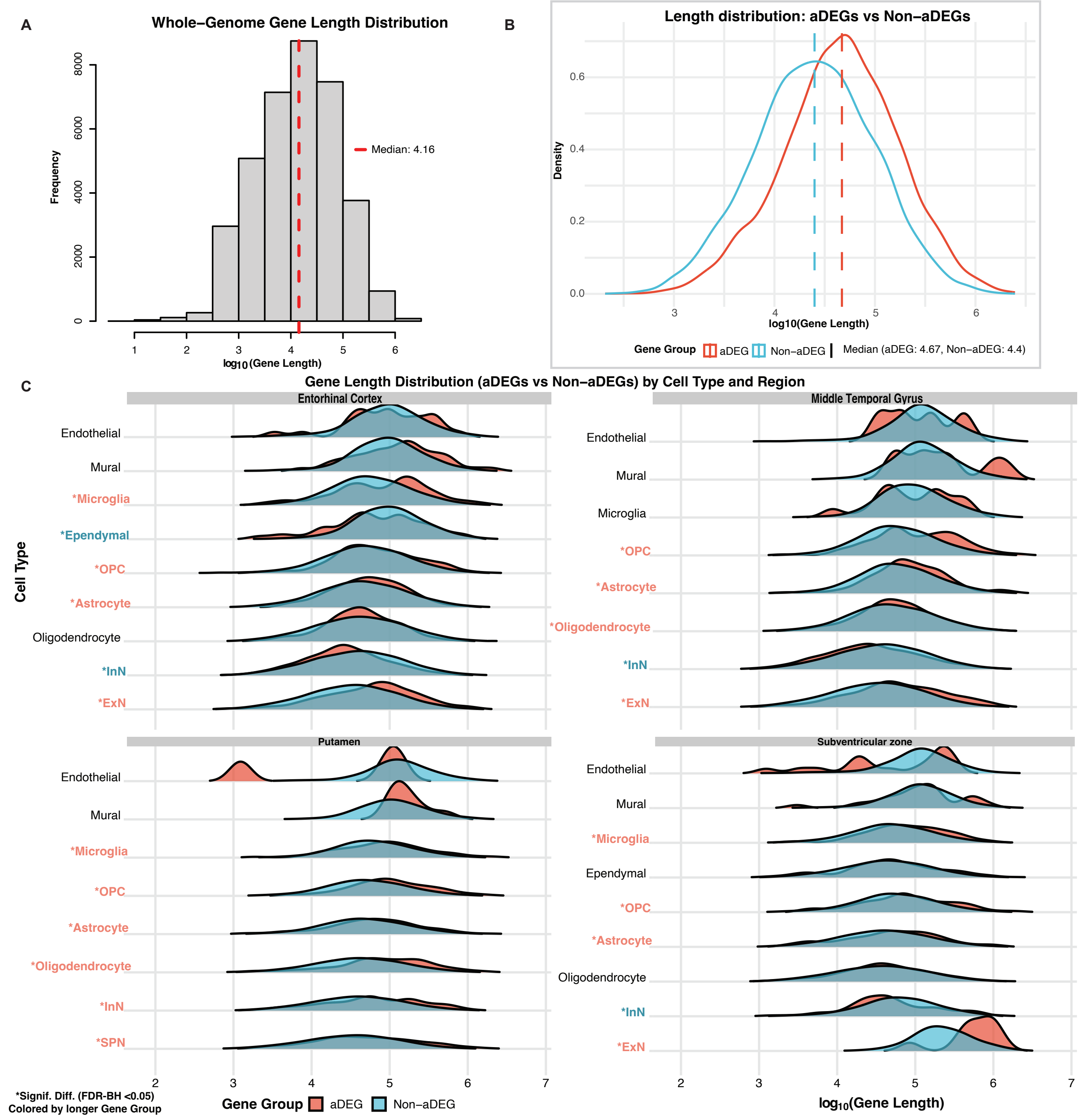

A
Whole−Genome Gene Length Distribution
Frequency
log10 (Gene Length)
Median: 4.16
B
Length distribution: aDEGs vs Non−aDEGs
Density
log10(Gene Length)
Gene Group
aDEG
Non−aDEG
Median (aDEG: 4.67, Non−aDEG: 4.4)
C
Gene Length Distribution (aDEGs vs Non−aDEGs) by Cell Type and Region
Entorhinal Cortex
Middle Temporal Gyrus
Putamen
Subventricular zone
Cell Type
Endothelial
Mural
*Microglia
*Ependymal
*OPC
*Astrocyte
Oligodendrocyte
*InN
*ExN
Microglia
*Oligodendrocyte
*SPN
Ependymal
*Signif. Diff. (FDR-BH <0.05)
Colored by longer Gene Group
Gene Group
aDEG
Non−aDEG
log10(Gene Length)

Supplementary Figure 6: Gene length of Age-Associated DEGs are longer than non-DEGS with variability across cell types and regions.

(a) Distribution of all gene lengths (in base pairs, determined via genomic-span, on log10 scale) across the whole-genome indicates a median length of 4.11. **(b)** Density of gene-length distribution (determined by genomic-span, log10 scale) of aDEGs (red, median: 4.67) as compared to non-aDEGs (blue, median: 4.4) across all cell-types and regions suggests that aDEGs tend to be longer than non-aDEGs overall (Welch's two sample t-test; $t_{15,842} = 29.914$, p_value < 2.2E-16). **(c)** Comparison of gene-length distribution (determined via genomic-span, log10 scale) between aDEGs (red) and non-aDEGs (blue) suggests variability in length differences across cell-type by region subsets with an overall trend towards aDEGs being longer than non-aDEGs (FDR-BH <0.05 indicated with bold font and asterisk; statistics provided in Supplementary Table 7).

Instances of Discordant aDEGs Shared between Regions

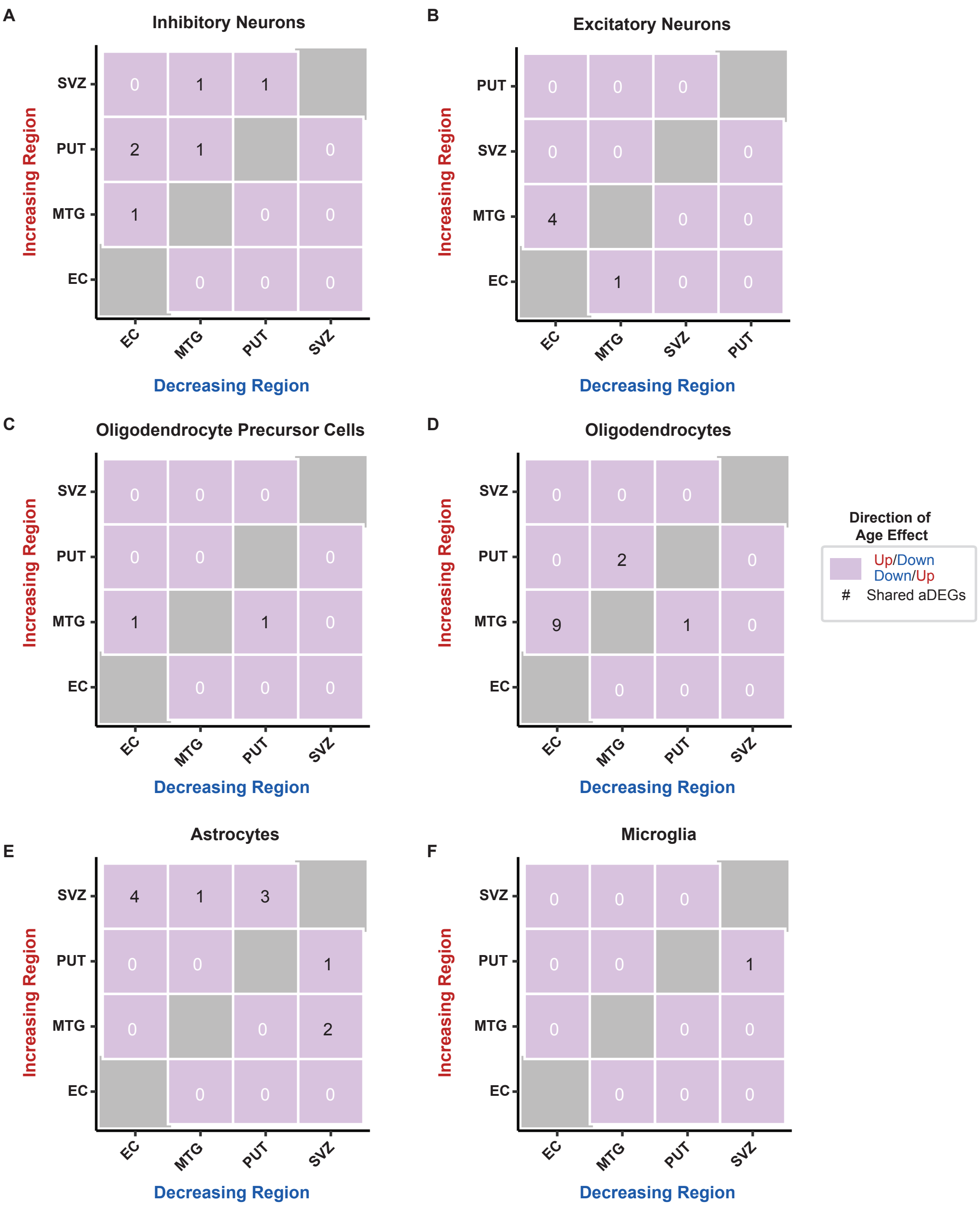

Supplementary Figure 7: Few regionally shared aDEGs with opposing age-effect directions across cell-types

Pairwise comparison of shared aDEGs that differ in age-effect direction between regions (discordant) within a given cell-type reveals few instances of discordant aDEGs.

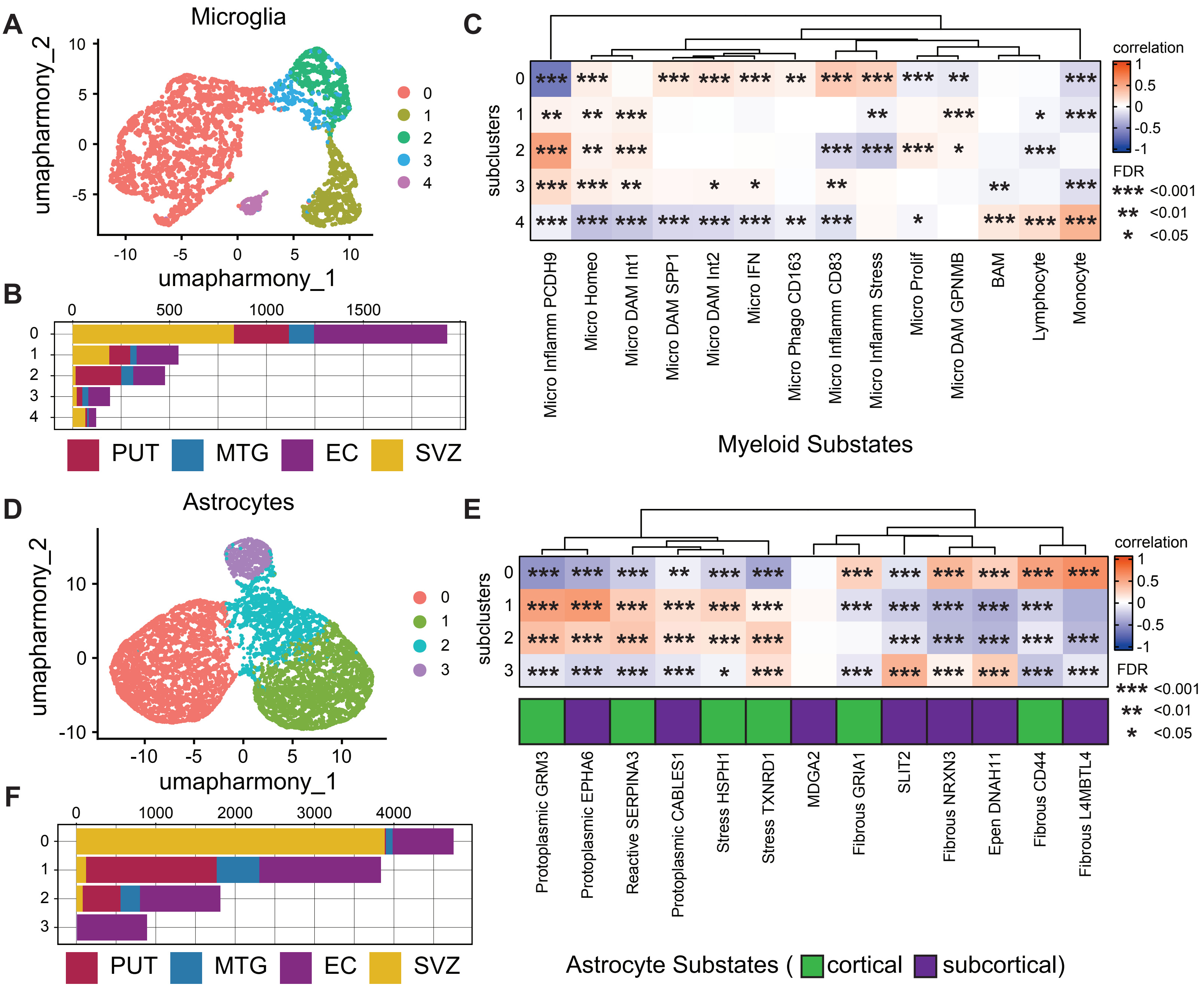

A
Microglia
umapharmony_2
umapharmony_1
0
1
2
3
4
B
PUT
MTG
EC
SVZ
C
correlation
FDR
*** <0.001
** <0.01
* <0.05
subclusters
Micro Inflamm PCDH9
Micro Homeo
Micro DAM Int1
Micro DAM SPP1
Micro DAM Int2
Micro IFN
Micro Phago CD163
Micro Inflamm CD83
Micro Inflamm Stress
Micro Prolif
Micro DAM GPNMB
BAM
Lymphocyte
Monocyte
Myeloid Substates
D
Astrocytes
umapharmony_2
umapharmony_1
E
correlation
FDR
*** <0.001
** <0.01
* <0.05
subclusters
Protoplasmic GRM3
Protoplasmic EPHA6
Reactive SERPINA3
Protoplasmic CABLES1
Stress HSPH1
Stress TXNRD1
MDGA2
Fibrous GRIA1
SLIT2
Fibrous NRXN3
Epen DNAH11
Fibrous CD44
Fibrous L4MBTL4
Astrocyte Substates (cortical subcortical)
F
PUT
MTG
EC
SVZ

Supplementary Figure 8: Subclustering of glia reveals states that are distinct from neurodegenerative disease-associated states.

Microglia (n = 3,265 across all donor x region combinations) and astrocyte (n = 11,289 across all donor x region combinations) subclustering performed in Seurat followed by average expression profile comparisons to known glial substates found across neurodegenerative diseases[27] performed in SAHA[139]. Spearman correlation of the normalized expression of top 2,000 variable features (respective to myeloid or astrocytes from Horan-Portelance and Acri et al.) performed for subcluster annotation. Significance bars represent adjusted p-values with the Benjamini-Hochberg algorithm. **(a)** Microglia subclustering reveals 5 subclusters ranging from 10 - 144 median cells per donor. (**e**) Microglia subcluster proportions by region. (**c**) Comparison to myeloid substates reveal one clear inflammatory substate (cluster 2 to 'Micro Inflamm PCDH9') and the presence of monocytes (cluster 4 to 'Monocyte'). (**d**) Astrocyte subclustering reveals 4 subclusters ranging from 2 - 306 median cells per donor. (**e**) Astrocyte subcluster proportions by region. (**f**) Comparison to astrocyte substates reveal a protoplasmic-fibrous delineation and one small subtype previously described to be specific to subcortical region(s) (cluster 3 to 'SILT+ astrocytes'). * p_adj < 0.05, ** p_adj < 0.01, *** p_adj < 0.001.

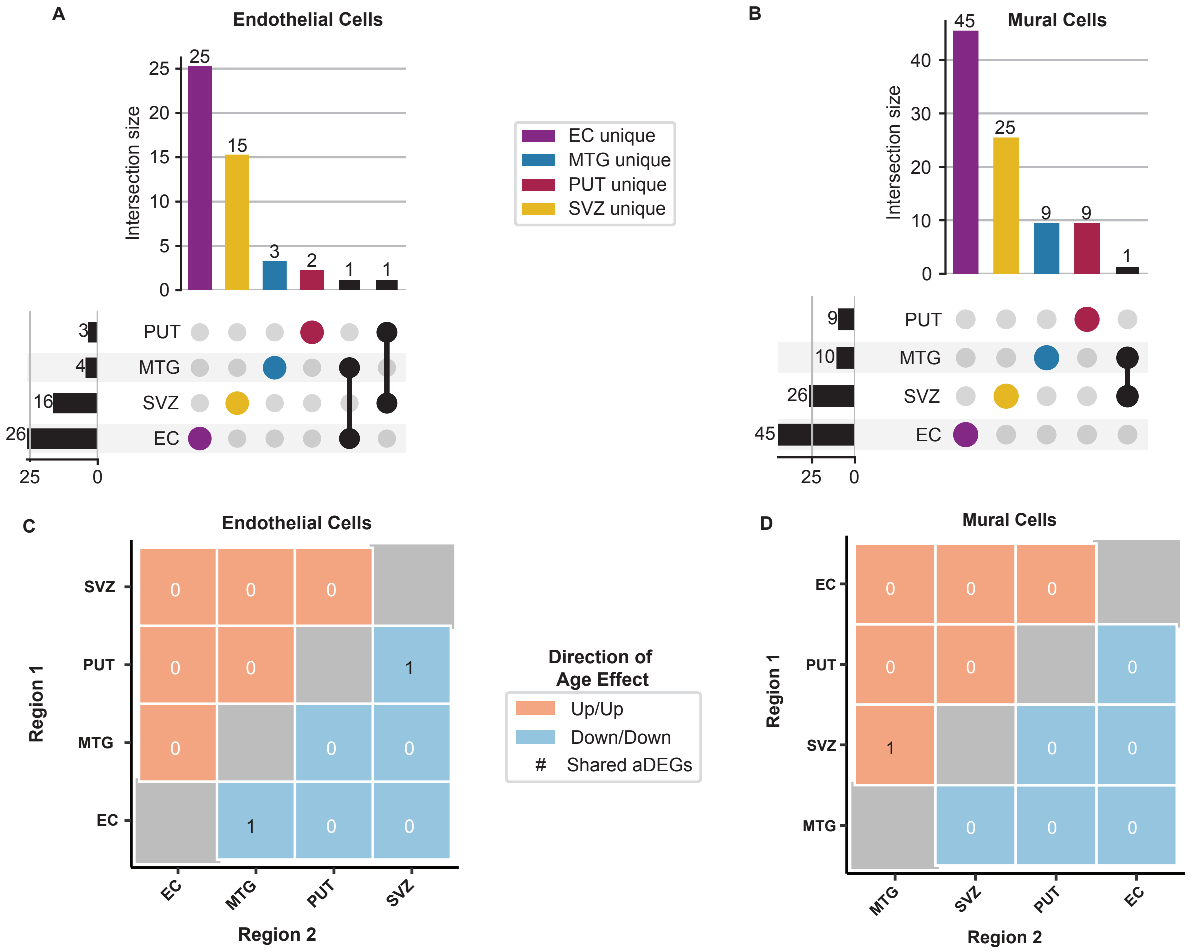
A
Endothelial Cells
Intersection size
25
15
3
2
1
1
PUT
MTG
SVZ
EC
3
4
16
26
25
0
B
Mural Cells
45
25
9
9
1
10
26
EC unique
MTG unique
PUT unique
SVZ unique
C
Endothelial Cells
Region 1
Region 2
SVZ
PUT
MTG
EC
Direction of
Age Effect
Up/Up
Down/Down
Shared aDEGs
D
Mural Cells

Supplementary Figure 9: Pericytes exhibit few aDEGs with the majority being in the EC and SVZ.

Regional distribution and overlap of pericyte cell-type aDEGs indicate that the majority of aDEGs are unique to a particular region. Both **(a)** endothelial cells and **(b)** mural cells have the majority of aDEGs localized to the EC followed by the SVZ. Pairwise comparison of shared aDEGs within pericytes suggests little regional sharing within both cell-types. Heatmap values representing count of aDEGs indicate the number of aDEGs changing in the same direction (concordance)--either increasing (positive, red) or decreasing (negative, blue)–in both of the indicated regions. **(c)** Endothelial cells showed 2 negatively concordant aDEGs one between the EC and MTG and the other between the PUT and SVZ. **(d)** Mural cells showed 1 positively concordant aDEG between the SVZ and MTG.

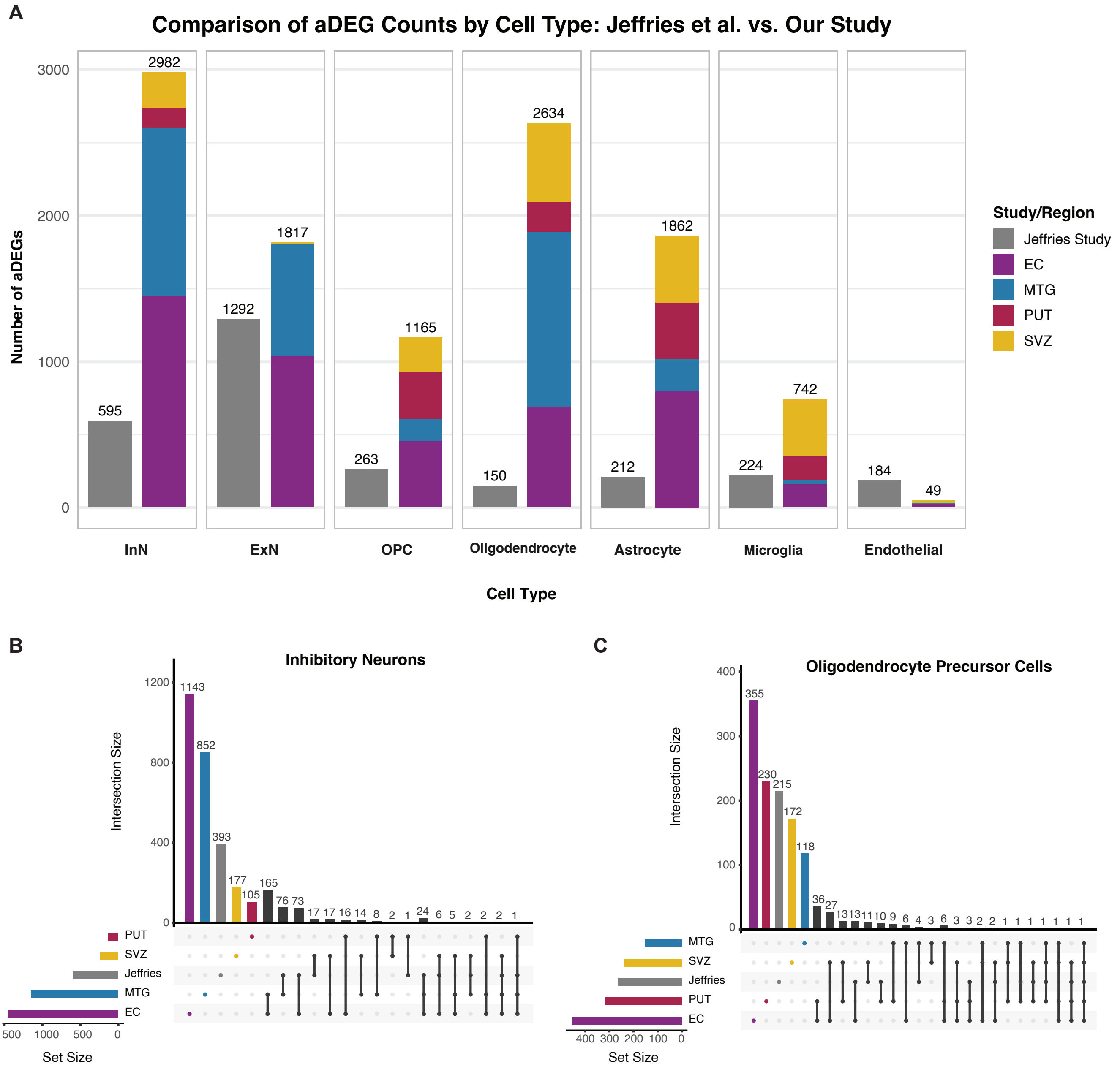
A
Comparison of aDEG Counts by Cell Type: Jeffries et al. vs. Our Study
Number of aDEGs
3000
2000
1000
0
2982
595
1292
1817
263
1165
150
2634
212
1862
224
742
184
49
InN
ExN
OPC
Oligodendrocyte
Astrocyte
Microglia
Endothelial
Cell Type
Study/Region
Jeffries Study
EC
MTG
PUT
SVZ
B
Inhibitory Neurons
Intersection Size
1200
800
400
0
1143
852
393
177
105
165
76
73
17
17
16
14
8
2
1
24
6
5
2
2
2
1
PUT
SVZ
Jeffries
MTG
EC
1500
1000
500
0
Set Size
C
Oligodendrocyte Precursor Cells
Intersection Size
400
300
200
100
0
355
230
215
172
118
36
27
13
13
11
10
9
6
4
3
6
3
2
2
1
1
1
1
1
1
1
MTG
SVZ
Jeffries
PUT
EC
400
300
200
100
0
Set Size

Supplementary Figure 10: Comparison to independent sample series from non-pathological prefrontal cortex confirms that aDEGs are likely largely region-specific. **(a)** Comparison of aDEG counts across broad cell types between Jeffries et al. and our study indicates a greater number of aDEGs found in our study. Distribution of **(b)** inhibitory neuron and **(c)** OPC aDEGs across studies/brain regions.

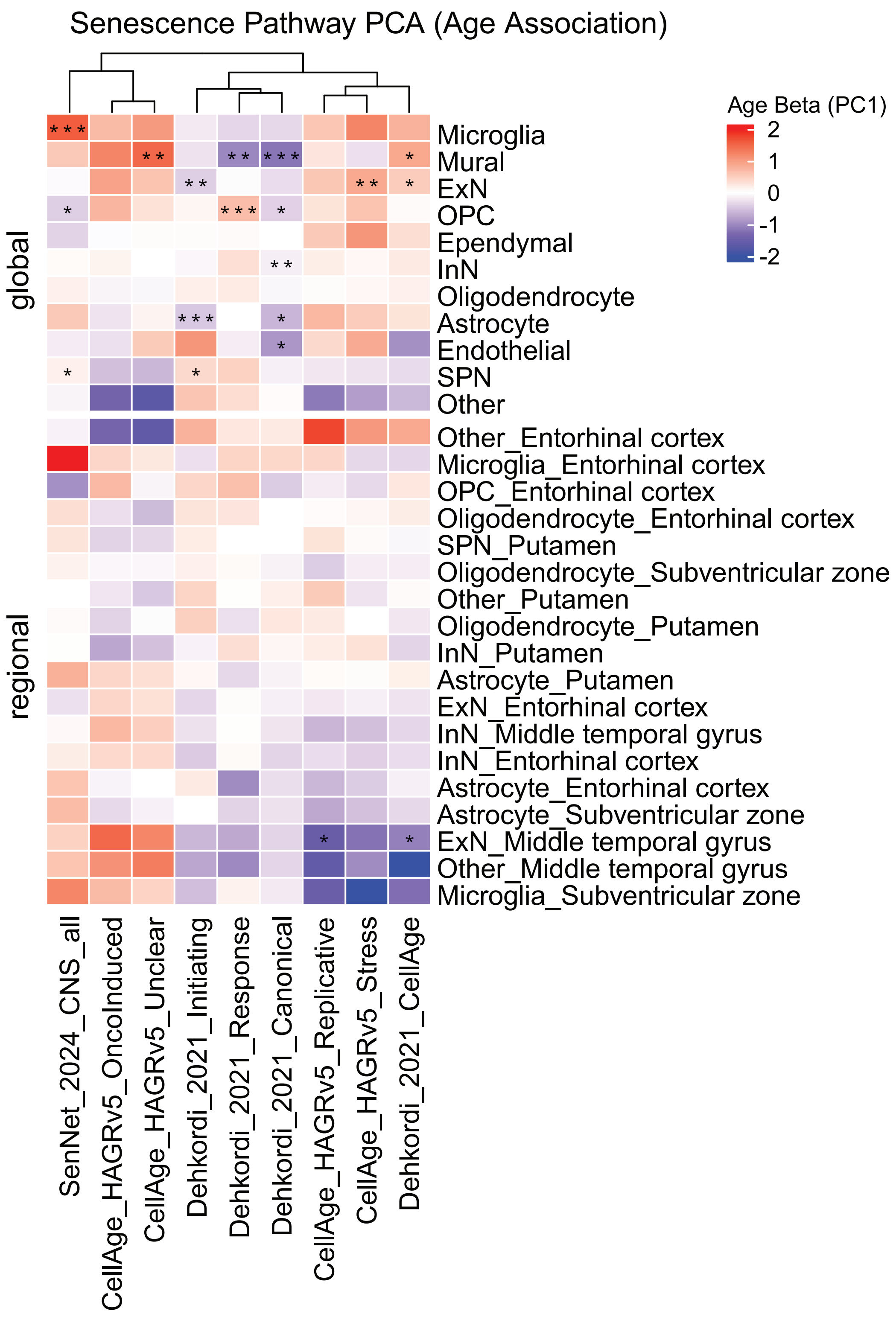

Senescence Pathway PCA (Age Association)
Age Beta (PC1)
2
1
0
-1
-2
global
regional
Microglia
Mural
ExN
OPC
Ependymal
InN
Oligodendrocyte
Astrocyte
Endothelial
SPN
Other
Other_Entorhinal cortex
Microglia_Entorhinal cortex
OPC_Entorhinal cortex
Oligodendrocyte_Entorhinal cortex
SPN_Putamen
Oligodendrocyte_Subventricular zone
Other_Putamen
Oligodendrocyte_Putamen
InN_Putamen
Astrocyte_Putamen
ExN_Entorhinal cortex
InN_Middle temporal gyrus
InN_Entorhinal cortex
Astrocyte_Entorhinal cortex
Astrocyte_Subventricular zone
ExN_Middle temporal gyrus
Other_Middle temporal gyrus
Microglia_Subventricular zone
SenNet_2024_CNS_all
CellAge_HAGRv5_OncoInduced
CellAge_HAGRv5_Unclear
Dehkordi_2021_Initiating
Dehkordi_2021_Response
Dehkordi_2021_Canonical
CellAge_HAGRv5_Replicative
CellAge_HAGRv5_Stress
Dehkordi_2021_CellAge

Supplementary Figure 11: Cell types from aged individuals engage senescence signatures across regions

Generalized linear model for the effect of binned age group on per-donor-per-celltype ("global") and per-donor-per-celltype-per-region ("regional") scores of senescence signatures (PC1_signature ~ Age_group; per cell-type across all regions). * FDR < 0.05, ** FDR < 0.01, *** FDR < 0.001 corrected across all comparisons (See Supplementary Data 16).

Supplementary Data 1: Donor demographics and tissue sample information
Per-donor level demographic information, including (from left to right): Sex (M=Male; F= Female); Age (in years); Age Category; (Young versus Aged); Diagnosis (abbrev. “Dx”, Control= no evidence of neuropathology); Ethnicity; Race; Smoking Status; Cause of Death; Highest level of education attained; Marital Status; Body Mass Index (abbrev. BMI); Postmortem Interval (abbrev. PMI, in hours); pH of brain tissue; APOE genotype; and tissue sample IDs for each available brain region (MTG = Middle Temporal Gyrus, EC=Entorhinal Cortex, PUT=Putamen, SVZ=Subventricular Zone). Donor and sample totals by age group, sex, and region are given in a summary table below the main table.

Supplementary Data 2: Nuclei counts and proportions by age group across regions, cell-types, and sexes
Tables reflecting both the number and proportion (percentage) of nuclei captured within both Young and Aged groups across regions (rows) and cell-types (columns). High level summaries of nuclei counts and proportions by sex within age groups are given off to the right-hand side.

Supplementary Data 3: Per-cluster level marker gene information
Per-cluster level marker gene information (Leiden algorithm, 0.85 resolution),

Supplementary Data 4: Per-cell-type level marker gene information
Per-cell-type level marker gene information (cell-type annotated Leiden clusters, 0.85 resolution)

Supplementary Data 5: Per-nuclei taxonomy
Per-nuclei information including (from left to right): pool name; sample ID; tissue source (brain bank); brain region of origin; clinical diagnosis; age (years); sex, lane number; channel; number of features; RNA counts; percent of mitochondrial DNA; group number; Leiden cluster number label (at 0.85 resolution);, age group; and broad cell-type level annotation.

Supplementary Data 6: Age-associated Differentially Expressed Gene (aDEG) information by cell-type
Workbook of aDEG information by broad cell-type (one per sheet). Includes information on age-effect, p-value, region of origin, Benjamini-Hochberg (FDR-BH) adjusted p-value, age-effect direction (1=upregulated; -1=downregulated), and gene type (protein coding vs lncRNA).

Supplementary Data 7: Region by cell-type level summary statistics for gene length comparison of aDEGs versus non-aDEGs
Region by cell-type level information and summary statistics for gene length comparison (in base pairs) of aDEGs versus non-aDEGs, including (from left to right): number of aDEGs, number of non-aDEGs, and median log-10 transformed length of aDEGs and non-aDEGs.

Lengths were compared using the Wilcoxon Rank-Sum test, and summary statistics include: W-statistic, p-value, and FDR-BH adjusted p-value.

Supplementary Data 8: Significant functional enrichments of each cell-type by region aDEG set

Workbook of significant (FDR-BH adj. P-value < 0.05) functional enrichments for each cell-type (one per sheet) by region aDEG set, as determined by *gprofiler2* package with customized background correction. Data provided includes: term size (number of genes comprising each pathway); query size (number of genes in cell-type x region aDEG set); intersection size (number of aDEGs that intersect with the pathway set); precision (intersections/term size); recall (intersections/aDEG set size); term ID (source database: # identifier); source database; term name; source-related information and identifiers; intersection (intersecting aDEGs); brain region, and broad cell-type.

Supplementary Data 9: Summary table of aDEG counts in Jeffries et al. versus our Study

Data provided includes cell- type (shared types only); study group; region (one of our four regions of interest or Jeffries PFC); and aDEG count for each cell-type x study x region group.

Supplementary Data 10: Per-cell type UpSet Plot inputs comparing Jeffries et. al. aDEGs to our aDEGs

Workbook with cell-type-specific sheets. Rows are gene names, columns are the Jeffries study (PFC region) and each of our four regions. A 1 indicates the presence of a gene in the given study/region whereas a 0 indicates absence.These matrices are formatted to serve as inputs for UpSet plot visualization of aDEG-sharing across studies/regions within a cell-type.

Supplementary Data 11: Per-nuclei demultiplexing information

Per-nuclei demultiplexing metrics including number of SNPs, number of reads, droplet classification (singlet, doublet, ambient), and donor source predictions and associated metrics. Select donor demographics and sample information (e.g. regional origin) are also included.

Supplementary Data 12: Per-cell-type UpSet Plot inputs to compare aDEG distribution and sharing across regions

Workbook with cell-type-specific sheets. Rows are gene names, columns are the regions of interest for our study (if present; MTG = Middle Temporal Gyrus, EC=Entorhinal Cortex, PUT=Putamen, SVZ=Subventricular Zone). TRUE indicates the presence of a gene in the given region whereas FALSE indicates absence.These matrices are formatted to serve as inputs for UpSet plot visualization of aDEG-sharing across regions within a cell-type.

Supplementary Data 13: Per-cell type HeatMap inputs to interrogate concordance of age effect direction of aDEGs shared pairwise between regions
Workbook with cell type-specific sheets. Rows are gene names, columns are the regions of interest for our study and their combinations (MTG = Middle Temporal Gyrus, EC=Entorhinal Cortex, PUT=Putamen, SVZ=Subventricular Zone). Down=decrease in expression with age; Up=increase in expression with age; NE=not expressed as aDEG in given region; dd = down in both regions (negatively concordant); uu=up in both regions (positively concordant); du=down in first region/up in second (discordant); ud=up in first region/down in second (discordant); duxx= down/up in one region and not expressed in the other.

Supplementary Data 14: Curated Senescence Pathways
Binary incidence dataframe (e.g. UpSet plot input) containing non-redundant senescence signatures from the NIH Cellular Senescence Network (SenNet), Human Ageing Genomic Resources database of cell senescence genes (CellAge), and senescence gene expression observed in human neurons (see Methods). Columns indicate which pathways genes are present in. The last column indicates whether the gene is one of the 2,000 highly-variable features used for clustering.

Supplementary Data 15: Senescence pathways present in cell-type markers
Fast Gene Set Enrichment Analysis (FGSEA, see Methods) output for enrichment of senescence pathway signatures tested against per cell-type ("test_name") marker gene sets.

Supplementary Data 16: Senescence pathways gene expression tested across age group
Generalized Linear Model (GLM) output for testing whether expression of senescence pathway genes were associated with age. Genes were tested per cell-type ("test") at both the broad cell-type ("global") and per-region ("regional") levels (see Methods).

Supplementary Data 17: Enrichment of senescence pathways in aDEGs
Dataframe examining overlap of cell-type x region aDEG set genes with senescence pathways. Results include cell-type ("ct"), region (MTG = Middle Temporal Gyrus, EC=Entorhinal Cortex, PUT=Putamen, SVZ=Subventricular Zone), cell-type x region aDEG set size ("de_size"), senescence pathway gene set size ("pathway_size"), senescence pathway, the intersection size ("overlap"), and Fisher's Exact test metrics (uncorrected p-value and FDR-BH adjusted).
